# Do federal and state audits increase compliance with a grant program to improve municipal infrastructure (AUDIT study): study protocol for a randomized controlled trial

**DOI:** 10.1186/1471-2458-14-912

**Published:** 2014-09-03

**Authors:** Ana L De La O, Fernando Martel García

**Affiliations:** Department of Political Science, Yale University, 77 Prospect Street, New Haven, USA; Cambridge Social Science Decision Lab Inc, 2020 Pennsylvania Ave, N.W Washington, USA

**Keywords:** Public services, Public health, Municipal governance, Accountability, Exploratory trial, Randomization, Hypotehsis testing

## Abstract

**Background:**

Poor governance and accountability compromise young democracies’ efforts to provide public services critical for human development, including water, sanitation, health, and education. Evidence shows that accountability agencies like superior audit institutions can reduce corruption and waste in federal grant programs financing service infrastructure. However, little is know about their effect on compliance with grant reporting and resource allocation requirements, or about the causal mechanisms. This study protocol for an exploratory randomized controlled trial tests the hypothesis that federal and state audits increase compliance with a federal grant program to improve municipal service infrastructure serving marginalized households.

**Methods/Design:**

The AUDIT study is a block randomized, controlled, three-arm parallel group exploratory trial. A convenience sample of 5 municipalities in each of 17 states in Mexico (*n*=85) were block randomized to be audited by federal auditors (*n*=17), by state auditors (*n*=17), and a control condition outside the annual program of audits (*n*=51) in a 1:1:3 ratio. Replicable and verifiable randomization was performed using publicly available lottery numbers. Audited municipalities were included in the national program of audits and received standard audits on their use of federal public service infrastructure grants. Municipalities receiving moderate levels of grant transfers were recruited, as these were outside the auditing sampling frame – and hence audit program – or had negligible probabilities of ever being audited. The primary outcome measures capture compliance with the grant program and markers for the causal mechanisms, including deterrence and information effects. Secondary outcome measure include differences in audit reports across federal and state auditors, and measures like career concerns, political promotions, and political clientelism capturing synergistic effects with municipal accountability systems. The survey firm and research assistants assessing outcomes were blind to treatment status.

**Discussion:**

This study will improve our understanding of local accountability systems for public service delivery in the 17 states under study, and may have downstream policy implications. The study design also demonstrates the use of verifiable and replicable randomization, and of sequentially partitioned hypotheses to reduce the Type I error rate in multiple hypothesis tests.

**Trial registration:**

Controlled-trials.com Identifier ISRCTN22381841: Date registered 02/11/2012

**Electronic supplementary material:**

The online version of this article (doi:10.1186/1471-2458-14-912) contains supplementary material, which is available to authorized users.

## Background

Many young democracies seem to be doing a poor job of delivering social services critical to human development, including water, sanitation, health, and education [1, 2]. One explanation is poor governance and accountability in public service provision [3–8]. In principle democracy causes rulers to act in the best interest of the majority, via periodic contested elections [9, 10]. In practice elections are blunt instruments of accountability [10–12]. Young democracies, in particular, often suffer from unstable party systems, lack of programmatic political platforms, deep inequality, ethnic tensions, and pervasive clientelism that compromise accountability [13, 14]. The task is further complicated by the magnitude of the challenge young democracies face, and the very nature of public service provision which includes long agency chains, multiple stake holders, hard to measure and verify multifaceted outcomes, and many tiers of management far removed from front line workers [1, 3]. Researchers have proposed accountability agencies as a useful institutional remedy capable of helping young democracies consolidate electoral accountability and improve service delivery [15, 16]. These are independent, non-elective, specialized bodies of oversight that provide relevant information on government performance, and sometimes sanction public officials on voter’s behalf [17–19]. Examples include election commissions, superior audit institutions (SAIs), anticorruption bodies, courts, human rights commissions, and statistical offices. Given young democracies’ weak electoral accountability, the magnitude of the tasks they face, and the complex nature of public service provision there is a manifest need for solid evidence on the effect of accountability agencies on public service delivery [10].

Evidence suggests SAIs are effective in reducing corruption and waste in public service infrastructure investment and in procurement of inputs. SAIs are external public auditors that monitor public expenditures and performance, often on behalf of the Legislature. For example, an increase in “audit intensity” put in place by the city of Buenos Aires reduced prices paid by local hospitals for basic, homogeneous inputs by 10–15 percent in the short term [20]. Experimental evidence has also shown how a 100 percent probability of an audit reduced missing expenditures in an Indonesian road construction project by some eight percentage points [21]. Another experiment in Brazil finds that “increasing audit risk by about 20 percentage points reduced the proportion of non-competitive procurement modalities adopted by local managers by about 17 percent [and] reduced the proportion of local procurement processes involving waste or corruption by about 20 percent” [22]. However, the experiment found no effect on the quality of publicly provided preventive and primary health care services, measured using client satisfaction surveys, nor on local compliance with national guidelines for the conditional cash transfer program Bolsa Família, measured in terms of beneficiary recruitment and enforcement of conditionalities. Additional evidence suggests the effectiveness of SAIs may be moderated by organizational features [23–26], and the degree of electoral competition in the polity [27]. These determine the objectivity, independence, and autonomy of the SAI. Experimental evidence also identifies synergistic effects between audits and municipal accountability systems [28–31].

There remain important gaps in this body of evidence. First, the mechanisms by which SAIs improve service delivery remains unclear. The economic approach to crime suggests wages, audit probabilities, and the degree of punishment deter dissonant behaviour by public employees and elected officials [32, 33]. But this ignores other causal channels, like knowledge acquisition by audited entities and changed perceptions about their administrative capacity. It also makes strong assumptions about the information and cognitive abilities available to agents. And it assumes negative audit reports will result in credible punishment, which is not always credible in young democracies. Second, most studies focus on the effect of SAIs on waste and corruption, yet SAIs can also ensure that services reach their intended beneficiaries by monitoring administrative compliance with national guidelines. Typically these stipulate what services are to be provided, how, and to whom. Third, the extant experimental evidence relates to marginal increases in the probability of audit and not the overall effect of the national program of audits (versus no program of audits). Besides, some experimental manipulations are unrealistic, like increasing audit probabilities to 100 percent. Fourth, some evidence points to synergistic effects between audits and municipal accountability systems [34] but whether these generalize to contexts where elected officials are limited to non-consecutive terms is an open question.

This study protocol for a block randomized, controlled, exploratory trial randomly assigns study municipalities in Mexico to be audited by federal auditors, by state auditors, and a control outside the national program of audits. It addresses three objectives: to identify the reduced-form impacts of randomized assignment to audits on outcomes such as knowledge about program requirements, compliance with the law and capacity building; as well as municipal governments’ spending priorities, and actual spending patterns. Second, to identify the reduced-form impacts of assignment to audit by either the federal or a state level SAI on audit verdicts, including the number of observations made, their severity, and the amounts of mandated reimbursements to federal treasury of misspent grant money. Third, to test for the effect of audits on career prospects, and on state governors’ discretionary allocations to municipalities. Table 1 provides list a pre-specified set of expected outcome hypotheses designed to meet these objectives.

**Table 1:** **AUDIT study hypotheses**

	Primary objective: Impact evaluation
H1	Municipal administrators in treated municipalities are aware of
	their treatment status
H2	Municipal administrators in treated municipalities audited in year 1
	believe the probability of being audited in year 2 is lower than in
	year 3
H3	Municipal administrators in treated municipalities have higher
	long-run beliefs about the probability of being audited
H4	Municipal administrators in treated municipalities have higher
	knowledge of FISM grant rules and regulations
H5	Municipal administrators in treated municipalities manifest
	preferences for municipal investments more in accordance with
	FISM priorities
H6	Municipal administrators in treated municipalities are more
	aware of lack of capacity and more likely to manifest plans for
	improving capacity
H7	Municipal administrators in treated municipalities are more likely
	to comply with FISM reporting and data accessibility rules
H8	Audited municipalities report allocating more FISM investment
	funds to localities outside the council seat and to public goods
** Secondary objective: Differences between state and federal audits**	
H9	The federal auditor (ASF) yields more observations and more
	refunds to the federal treasury than state auditors (EFSL)
H10	The ASF yields more severe observations and opinions than EFSL
** Tertiary objective: Interactions with local accountability system**	
H11	Municipal administrators in treated municipalities have different
	expectations about future political appointments
H12	Municipal administrators in treated municipalities have different
	expectations about career prospects
H13	Municipal administrators in treated municipalities perceive the
	ASF as a more important principal
H14	State governors compensate audited municipalities for refunds to
	the federal government

This table lists the pre-specified hypotheses that will be tested to help meet the study objectives.

### Policy context

Mexico’s municipalities provide basic public services like drinking water, sanitation, improved road surfaces, and electricity, to 113 million citizens, though access to these services remains uneven across, and within, Mexican municipalities. Improving access of marginalized populations to basic municipal public services is a key element of Mexico’s National Development Plan 2007–2012 [35]. The main instrument available to the Federal Government to achieve this goal is public spending, including earmarked federal grants. For example, the federal Contribution Fund for Social Infrastructure (FISM, in Spanish) provides grants for municipal investments in basic public service infrastructure benefiting local marginalized populations. In FY 2009 it financed one-third of all basic public investment in municipalities, or some 100,000 individual investments [36]. However, the reliance on federal transfer schemes as the key instrument for improving access to public services is not without risks. Municipalities’ ability to identify marginalized communities, diagnose their basic public service needs, propose policy solutions, and implement them is weak. Moreover, the use of federal funds for purposes unrelated to the development of marginalized areas, embezzlement, and corruption are a problem [36–38]. The principal mechanism by which the Federal Congress oversees local governments’ use of federal resources is the national program of audits, directed by the Superior Federal Auditors (ASF, in Spanish) in coordination with the Superior Audit Entities of States (EFSL, in Spanish).

The AUDIT study explores the role that audits play in local accountability systems for infrastructure investments financed by the FISM grant program. The study is based on a field experiment we conducted in partnership with Mexico’s Superior Federal Auditor.

## Methods/Design

### Trial design

The AUDIT study is a block randomized, three-arm parallel group, exploratory trial on a convenience sample of 85 municipalities in Mexico. Blocking was done by state across 17 states, with five municipalities per block. Using non-uniform random assignment and a 1:1:3 blocking ratio we assigned one municipality per block to be audited by the ASF, another by the EFSL, and the remaining three municipalities to the control condition (no intervention). Our reporting of the trial design follows the CONSORT 2010 Checklist [39, 40] (See Additional file 1). The trial received an ethics approval by Yale University’s Human Subjects Committee (ref: 1106008610), and is registered with the International Standard Randomised Controlled Trial Number Register (ISRCTN22381841) and the Experiments in Governance and Politics Network (No:20121031). All end line survey participants are required to give informed consent.

### Participants

Inclusion criteria for participation were designed so as to minimize disruption to the Annual Program of Audits directed by Superior Federal Auditors (ASF, in Spanish) in coordination with the Superior Audit Entities of the States (EFSL, in Spanish) [41]. The study focuses on audits of municipalities’ use of grants from the federal Contribution Fund for Social Infrastructure (FISM, in Spanish). This fund provides grants for municipal investments in basic public service infrastructure benefiting local marginalized populations. The ASF determines which federal programs and recipient entities will be audited and, with regards to FISM related audits, it can also choose to perform the audit itself or request the relevant state EFSL perform it. Against this background the specific inclusion criteria are as follows:

*Stage 1* From the universe of 2,440 municipalities located in 31 states select:

1. States with more than 20 municipalities;
2. Municipalities with FISM transfers in 2010 of 10 million pesos or more;
3. Municipalities not audited in the previous two years (2009, 2010);
4. Municipalities not amongst the 43 pre-selected by the ASF for the 2011 National Program of Audits.

***Stage 2*** From this selection of 767 municipalities located in 21 states select:

1. States with 5 or more municipalities;
2. For each state, rank municipalities in decreasing order of FISM transfers and choose by state the five municipalities with ranks 6 to 10.

The first stage of the selection process of our convenience sample guarantees that our experimental sample includes municipalities that are of relevance to the ASF in terms of the amount of transfers received through the FISM transfer scheme. The second stage of the selection process ensures we have 5 municipalities per state in the experimental group; that our experimental group includes municipalities that are unlikely to have been audited since 1998, when the current audits to FISM expenditures began; and that, within states, municipalities in our sample are similar in terms of the amount of transfers received through the FISM scheme. The final selection includes 5 municipalities in each of 17 states for a total experimental group sample of 85 municipalities. Municipalities that did not meet these inclusion criteria were excluded.

### Randomization and interventions

We use a verifiable and replicable block randomization procedure based on publicly available state lottery numbers. The chosen method had to meet two major constraints. First, it had to be sufficiently simple that the ASF could explain, justify, and replicate the randomization mechanism to Congress. Second, the randomization process had to be compatible with the operational and technological infrastructure of the implementing agency (effectively limiting software solutions to Microsoft Excel). The experimental group consists of 17 blocks with 5 municipalities each. Using non-uniform random assignment and a 1:1:3 blocking ratio we assigned one municipality per block to be audited by the ASF, another by the EFSL of the block’s state, and the remaining three municipalities to the control condition (no intervention). Specifically the block randomization process proceeded as follows:

- By state, we provided each municipality with a pair of single-digit “tickets”:
- Block municipalities by state
- In Excel list municipalities in increasing order based on their individual identifier provided by the Mexican National Institute of Statistics and Geography (INEGI, in Spanish).
- Assign each municipality two single-digit “tickets”, and do this sequentially for all municipalities (e.g. 0-1, 2-3, 4-5, 6-7, 8-9 …).
- We generated a random vector of “winning digits”:
- To generate the random “winning digits”, we used the winning numbers of the seven largest prizes of the Mexican National Lottery of the first Tuesday of March 2011.
- Each winning number has 5-digits.
- We ordered the 5-digit winning numbers in decreasing order of prize.
- Our first five “winning digits” come from the number associated with the highest prize (e.g. for the date we used, the number was 23862 and the price 5 million pesos), the next ten “winning digits” digits come from the second and third prizes.
- The fourth largest prize (of 80,000 pesos) was won by four numbers. To order these tied lottery numbers randomly, we (1) ordered the numbers in increasing order; (2) grab the number associated with the largest prize in the lottery of 22 February (e.g. number 36625), delete one repeated digit (e.g. becomes 3625); (3) assign one of these digits to each of the four tied lottery numbers; (4) use this assigned digit to sort the four tied lottery numbers in increasing order (e.g. 2,3,5,6).
- Concatenating the 15 “winning digits” from three lottery numbers associated with the three top prizes, and the random ordering of the four lottery numbers tied for fourth prize, gives us a random vector of 35 “winning digits”, enough to randomly assign 17 municipalities to ASF audit, and 17 municipalities to EFSL audit.
- We then assigned municipalities to treatment arms based on the random vector of “winning digits”:
- Start reading from the top of the vector of “winning digits”. The first winning digit is a 2, so assign the municipality in the first state holding the single-digit “ticket” 2 to an ASF audit. Then, use the second “winning digit” from the vector to assign a municipality in the second state to ASF audit, and so on for all 17 states.
- Repeat the procedure – starting from the 18^th^ element of the vector of winning digits – to allocate one municipality by each of the seventeen states to an audit by the EFSL.
- Municipalities not allocated to EFSL or ASF serve as control.

A worked example of the randomization procedure is provided in Table 2. The process of randomization was carried out by the researchers (AO and FM) and approved and implemented by the ASF in collaboration with the EFSL.

**Table 2:** **Example of random allocation for two states**

PANEL A: generating the random allocation sequence of “Winning Digits”						
**Lottery 3/1/2011**		**Lottery 2/22/2011**				
**Number**	**Prize (millions)**	**Number**	**Prize (millions)**			
23862	5	36625	5			
19186	0.4					
54595	0.2	Sort order (ascending)				
02437	0.08	3				
09502	0.08	6				
42585	0.08	2				
45776	0.08	5				
**PANEL B: randomization of municipalities**						
**ID**	**State**	**Municipality**	**FISM transfer (millions)**	**“Ticket” Digits**	**ASF**	**EFSL**
07022	Chiapas	Comitán de Domínguez	69	0-1		
07028	Chiapas	Chenalhó	61	2-3	1	
07076	Chiapas	Ocozocoautla de Espinosa	68	4-5		1
07092	Chiapas	San Cristóbal de Las Casas	65	6-7		
07111	Chiapas	Tecpatán	59	8-9		
08012	Chihuahua	Carichí	12	0-1		
08021	Chihuahua	Delicias	15	2-3	1	
08030	Chihuahua	Guazapares	11	4-5		
08032	Chihuahua	Hidalgo del Parral	13	6-7		
08066	Chihuahua	Uruachi	13	8-9		1

The sequence of random numbers from Panel A is: 23862, 19186, 54595, 42585, 02437, 45776, 09502. We use the first 17 winning digits to allocate one municipality by state to an audit by ASF. For example, the first winning digit in the random sequence is a 2. Because Chenalhó was allocated that “ticket” (see Panel B, Digits column), it is selected to be audited by ASF. The second winning digit in the random sequence is a 3, and so Delicias is selected, and so on for the remaining 15 states. To allocate EFSL we begin at the top again, starting with the 18^th^ digit in the random sequence, a 5. Accordingly, Ocozocoautla de Espinosa is allocated to EFSL, and so on. Had the 18^th^ digit been a 2 or a 3, we would have skipped that digit, moved to the next digit different from 2 or 3, and used that digit to allocate the first municipality to EFSL. One municipality cannot be assigned to both ASF and EFSL.

**Figure 1:**
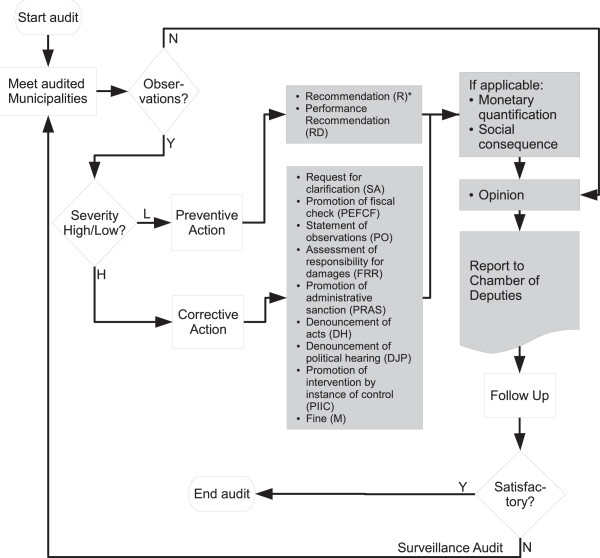
**Flow chart of Superior Federal Auditor’s audit process.** Flow chart depicting the Superior Federal Auditor’s (ASF) audit process of municipal expenditures under the federal Contribution Fund for Social Infrastructure (FISM) grant program [43]. Highlighted in grey are ASF judgements, opinions, and outputs.

The method of randomization adopted is transparent, replicable, and verifiable. In addition, the only software requirements are a web browser (to access the lottery numbers) and Microsoft Excel. These features were key for the ASF to accept the procedure. However, the lottery numbers span the range 00000 to 59999. Accordingly, the first digit of every winning lottery number can only take the values 0 through 5 while all other digits that can take values from 0 to 9. Thus, the fourth and fifth municipalities in the first state of our study have in practice zero chance of being audited by the ASF because they hold “tickets” (6,7) and (8,9) respectively. After the first assignment, this happens every fifth assignment, when a new lottery number is added to the sequence of “winning digits”. In other words, the randomization procedure generates known non-uniform probabilities of treatment in a subset of the blocks. Only 4 assignments to ASF and 3 to EFSL are affected by the non-homogeneous randomization. Even so, because the probabilities of assignment are known exactly we can adjust randomization hypothesis tests and use inverse probability weighting for estimates. Municipalities assigned to an audit are audited as usual by the assigned federal or state auditor [42]. Figure 1 provides a schematic layout of a municipal FISM audit process.

### Outcome measures

The primary outcome measures of this study capture the effectiveness of the national program of audits amongst the study group. Primary outcomes follow an expected causal order, going from how audits may affect subjects’ beliefs about future audits, to how they modify subjects’ knowledge of program rules, investment preferences, awareness of capacity limitations, compliance with reporting requirements, and the actual allocation of investments between outlying settlements and the council seat (see Table 1). Secondary outcomes compare the effectiveness with which the federal and state level auditors uncover wrongdoings; the severity with which they judge them; and the diligence with which they pursue wrongdoings. (If solid evidence of differences is found, we will do some additional exploratory work, like subgroup analysis by stratifying on the basis of an institutional quality index [44]). Tertiary outcomes explore possible interactions between audits and local accountability systems. We do so by comparing how audits may affect subject’s expectations about future political appointments, career prospects, perceive their principals differently, and whether state governors engage in clientelist practices to blunt the effect of audits on municipalities of their same political persuasion. Due to their specificity most outcome measures were defined and measured by the investigators using a proprietary survey, and related measurement instruments. Specific definitions, measurements, and sources are described in Additional file 2.

Our outcome data come from routine audit reports, other official sources, direct observations by the investigators, and from a proprietary survey of municipal administrators. The survey was developed by the investigators and implemented by the Mexican survey firm *Data Opinion Publica y Mercados*. The survey firm was blind to treatment status. The survey was pilot tested on four municipalities similar to the ones in the experimental group, and the results where used to clarify the meaning of questions and adapt the length of the survey, as well the contact strategy. The survey was fielded over the phone between April 27, 2012 and June 7, 2012. We administered the survey to key personnel in each municipality, including: the Municipal President, Treasurer, Director of Public Services, Director of Public Works, and/or Director of Urban Planning. It was not always possible to contact the personnel, in which case we moved down the municipal hierarchy. Given the sample size of this study, strenuous efforts were made to ensure full response. A copy of the survey is included in Additional file 3. Data from official sources will be collected by a research assistant according to guidelines provided by the researchers. Some data will be collected through direct observations (e.g. does municipality have a web page) according to a measurement instrument developed by the researchers and implemented by a research assistant. Collection of these data is expected to end on January 30, 2013. The research assistant is blind to treatment status. Finally, most outcomes of interest are subjective in nature. This introduces some well known limitations.

### Sample size

No power analysis was done for this field experiment. First, our implementing partner (the ASF) gave us a strict limit on the number of audits they would allow us to randomize. Second, a power calculation would have been complicated by the number of primary outcomes in this exploratory trial. Third, not enough data from relevant prior studies were available to inform the statistical sample size calculation. Given these restriction we powered the study by using an unbalanced block design, which improves covariate balance and efficiency. The only limit on the number of controls was our own budget, and concerns for bias if the study became too unbalanced. Hence the sample size was determined *a priori* to 85 municipalities. Finally, blocks with four or more units may have some advantages relative to pair matching [45, 46]. As an additional check we will do *ex post* power calculations for minimum detectable effect sizes for key outcomes.

### Blinding

Whereas researchers and ASF management in Mexico city are fully aware of treatment allocations, the survey firm and research assistants collecting outcome data were kept blinded to the allocation. The researchers took no specific measures to ensure field auditors carrying out the audits were blinded to the allocation. Similarly, municipal staff are clearly aware whether they are being audited or not, but there is not reason to expect them to know they are part of an experiment. Finally, because the researchers are not blind to the allocation they will carry out the data analysis according to the detailed analytical plan in Additional file 2.

### Statistical methods

Because our sample is relatively small and we are concerned about power our approach is to start by asking very little of the data, and then ask progressively more depending on the answers to previous queries. The inferential framework is as follows:

1. *Sharp null hypothesis test*: We begin by testing the sharp null of no effect on any unit against the alternative of some effect (e.g. change in location, scale, or distribution). These tests can tell us whether the treatment has an effect, but they are silent as to the magnitude and variability of the effect.
2. *Visual inspection of outcome distributions*: We plot histograms, box plots, and density plots, as befits the type of measurement, for the outcomes of interest across treatment arms.
3. *Descriptive inference*: We describe measures of central tendency, like experimental group averages and their standard deviation, along with the difference across averages and their standard deviations (so-called ATEs). For the latter we ignore the covariance term in Var (*Y*_*C*_−*Y*_*T*_)= Var (*Y*_*C*_) + Var (*Y*_*T*_)− 2 Cov (*Y*_*C*_,*Y*_*T*_) as it is not observed, where *Y* is the outcome of interest and subscripts refer to treatment and control conditions. This provides a more conservative estimate.
4. *Modeling*: To generate estimates of causal effects and confidence intervals we need to assume non-interference and a model of causal effects. We check the nature of the underlying model assumptions by performing model diagnostics including testing normality of residuals, homoscedasticity, plotting residuals against predicted outcomes, and comparing the actual experimental data to fake data generated from the estimated model [47, 48].

Because the treatment was randomized with known probabilities we rely on randomization tests of the sharp null of no effect on any unit [49]. The specific randomization statistic chosen will be appropriate to the category and distribution of the outcome measures. We will use sequential partitioned hypothesis testing to address the multiplicity of analyses and outcomes and control the Type I error rate [50, 51]. We will let exploratory data analysis and model checking determine whether we model the outcome by inverting randomization tests or via robust OLS estimation, though our default is to rely on additive effects and inversion of sharp null hypothesis tests (see Annex A). Finally, whereas the treatment was randomized to municipalities, some outcome variables are measured at the level of individual municipal administrators. At this level the treatment can be thought of as cluster randomized. We will analyze these data at the individual level and check for robustness by comparing inferences to a differences in total outcomes estimator and to aggregating individual level at the municipal level [52]. A detailed analytical plan is available as Additional file 2.

### Funding

The AUDIT trial is generously funded by the Institution for Social and Policy Studies and the Leitner Program in International and Comparative Political Economy, both at Yale University, and by New York University’s Department of Politics.

## Discussion

Randomized control trials are not immune from numerous threats to inference including attrition, non-compliance, and measurement error.

### Attrition

Attrition and missing outcomes can undo the benefits of randomization as observed outcomes may no longer be representative of the full experimental population nor comparable across observed experimental arms [53]. Due to small sample size we tried to prevent attrition by intensive follow up of non-respondents. We also collected logs of call efforts from the survey firm, under the assumption that those hardest to reach are similar to those never reached. We will also try to fill in missing response covariates (e.g. age, gender, and career history of of municipal official) using publicly available information. At the analytical stage we will do the following:

1. Diagnosis: We will report the prevalence of attrition across experimental arms and check the covariate profiles of units missing outcomes versus those reporting outcomes. We will also check how observed outcomes vary with the recorded logs of call efforts.
2. Hypothesis test: We will test the sharp null of no effect of treatment on attrition. Failure to reject the null that the treatment has no effect on the attrition strongly suggests that the observed units are at least comparable across treatment arms [53].
3. Imputation: If the null is rejected then a complete data analysis is only appropriate if the outcome does not cause attrition and the only cause in common between the outcome and the attrition is the treatment [53]. This is a strong assumption. For robustness we will draw inferences using extreme bounds, and consider trimmed bounds, multiple imputations and inverse probability weights analyses as secondary analyses.

### Non-compliance

Non-compliance arises whenever experimental units receive a treatment different from the one assigned to them, and it can undermine the benefits of randomization [54]. For example, we know two municipalities could not be audited because of drug related violence. In addition, our partnership with the ASF allowed us to randomize the schedule of audits under the National Audits Program but EFSLs may choose to perform additional audits outside this program, though we do not expect two-sided non-compliance to be extensive. Because EFSLs report the complete list of municipalities they audit to the ASF so we will know the actual treatment status of all municipalities. To account for two-sided non-compliance we will proceed as follows:

1. Using the treatment assignment variable test the sharp null of no effect (e.g. intention to treat analysis). If no null is rejected stop and declare the null of no treatment effect cannot be rejected. Otherwise proceed to estimation of effects.
2. Estimate the ITT effect and, assuming monotonicity, the effect of treatment on the treated (ETT) using a permutation approach to instrumental variables [55]. (The latter is chosen for convenience as it is better adapted to dealing with the non-homogeneous randomization. If non-compliance is two-sided we will estimate the effect on compliers only).
3. Report non-parametric natural bounds on the ATE [56].

### Interference

Interference occurs when outcomes for any given unit depend, not only on its own treatment status, but also on the profile of treatments for others units in the experimental group. In the extreme case where control units benefit as much as the treated units from a given treatment profile the estimated ATE will be zero even though the treatment might have been hugely beneficial. There is an effect but no primary effect (conditional on interference) [57]. To test for the presence of interference and control for it we need to assume a model of interference. In our discussion with employees of the ASF we learned that municipal officials talk to each other with regards to the audit program. We will assume talking is along party lines and limited to other municipalities in the same state (parties are organized around states). (Geographic distance between municipalities may not be that important considering the degree of cell phone and email penetration in Mexico but we might consider it in a secondary analysis). We will also assume that the intensity of talking depends on the similarity of the municipalities, as they are more likely to have interests in common. We will proxy for similarity using FISM grant amounts. These are decided by a formula (and some gubernatorial discretion) that takes as inputs socio-economic indicators. We check for interference using municipalities outside the experimental group (their exposure is random [52]) using administrative data from the Federal Treasury detailing what categories of municipal public goods municipalities invest in and their rate of disbursements. Specifically we proceed as follows:

1. We define the distance measure for municipality *i* in experimental state *j* as , where *x*_*ij*_=1 if at least one of the audited municipalities in state *j* has a major with the same party affiliation as municipality *i*, and where  is the amount of FISM transfers (*w*_*ij*_) received by municipality *i* in state *j* as a fraction of the average transfer received by audited municipalities of the same party affiliation () in the same state *j*. If none of the audited municipalities share a party affiliation we set *y*_*ij*_=0. To ensure *y*_*ij*_∈ [ 1,0) we only calculate the measure for municipalities that receive same or lower transfers than those in the experimental group.
2. Since our distance measure is continuous, we stratify municipalities into quartiles defined by *y*. Along with the binary *x*, this defines a 4×2 table of outcomes, where one column is units treated with spillover effects of magnitude *y*_*q*_ and the other column is assumed to receive no spillover.
3. As noted, dependent variables will be derived from the PASH files which cover almost all municipalities in Mexico. These include whether municipalities report to the Federal Treasury, what categories of municipal public goods they invest in, and the rate of disbursements among other.

Given the definition of the distance measure and the fact that experimental municipalities are also blocked on *y* finding strong evidence of spillover effects would severely compromise the detection of ATE within the experimental group using the survey data. That said, we can proceed as above and define *d*_*ij*_ for each municipality in the experimental group (by definition treated municipalities score a 1). Since these have already been blocked on *y* most of the variation – if any – will come from the party affinity measure within the block. As usual we can proceed by testing a family of sharp nulls where we classify as treated all municipalities with *d*_*ij*_>0 and control otherwise. Rejecting the sharp null would suggest treatment and its spillover has an effect. If so we can further test the no null of no effect between treated units and those subject to spillover by defining treated as those with *d*_*ij*_=1 and control as those with 0<*d*_*ij*_<1. For estimation we use inverse probability weights [52].

In conclusion, the block randomized, controlled, three-arm parallel group exploratory AUDIT study on a convenience sample of 85 municipalities in Mexico fulfills standard scientific criteria for evidence-based evaluation [58], and reporting (see Additional file 1). We are confident the aforementioned measures to deal with threats to inference will be sufficient to ensure the AUDIT study will meet its objectives. Namely, to assess the efficacy of the national program of audits in improving compliance with a federal grant program to improve municipal infrastructure. And to explore the mechanisms by which any effects take place; the influence of institutional differences; and potential synergies with local accountability systems. Finally, the study design also demonstrates the use of verifiable and replicable randomization, and of sequentially partitioned hypotheses to reduce the Type I error rate in multiple hypothesis tests.

## Trial status

The AUDIT study is currently analyzing the outcome data (this protocol was first submitted for publication in January 2013).

## Electronic supplementary material

Additional file 1: **CONSORT 2010 Checklist.** (PDF 209 KB)

Additional file 2: **Detailed analytical plan.** (PDF 526 KB)

Additional file 3: **Survey instrument.** (PDF 92 KB)

## Abbreviations

ASF (in Spanish): Superior federal auditor
EFSL (in Spanish): Superior audit entities of states
FISM (in Spanish): Contribution Fund for Social Infrastructure
SAI: Superior audit institution.
