# Supplementary material for: Do federal and state audits increase compliance with a grant program to improve municipal infrastructure (AUDIT study): study protocol for a randomized controlled trial
Source: BMC Public Health. 2014 Sep 3;14:912. doi: 10.1186/1471-2458-14-912 (PMC4175219; doi:10.1186/1471-2458-14-912)
Supplement: Supplementary file 2 — Additional file 2: Detailed analytical plan. (PDF 526 KB) [file 12889_2013_7060_MOESM2_ESM.pdf]

A Detailed Analytical Plan

A.1 Primary objective: Impact evaluation

This section focuses on those aspects of municipal behaviour of concern to the SAI, namely whether municipalities comply with the various aspects of the law governing the federal infrastructure grant program.

A.1.1 Hypotheses about awareness of treatment status

In this section we ask whether experimental units at endline are aware of their treatment status, and attempt to identify the possibility of short- and long-term effects of intervention. First, units exposed to treatment, which included being audited by either the federal or state level auditors (ASF or EFSL respectively), ought to be aware they were audited the previous year. However, this might not be the case if there was a change in administration in the period between treatment and survey collection. Alternatively, the same administration may remain in place, but key personnel may have changed. Hence, responses may be moderated by whether personnel answering the survey were in place during the previous audit. Indeed, frequent changes in personnel are often seen as a problem in municipal administration.

Outcome Concepts

Awareness of treatment status by relevant municipal administrators.

Measurement Instrument

Audit Module, Municipal Administration Survey (see Appendix B).

Outcome Measures

Municipal administrators' awareness scale (Y_{ij}): $Y_{ij} = \sum_{q=1}^{5} R_{qij}$ is the sum score of observed binary responses R, to questions 1 through 5 in the relevant measurement instrument by municipal administrator $i \in$ {Municipal President, Treasurer, Director of Public Services, Director of Public Works, and/or Director of Urban Planning}, in municipality j. If the answer is correct $R_{qij} = 1$, and if the answer is incorrect or the subject does not know $R_{qij} = 0$. Higher scores indicate higher awareness.

Analytical Plan

After rank alignment, we expect audits by the federal auditors (ASF) to have a bigger effect than audits by state-level auditors (EFSL).

Step 1 $H_0^{1.1} : \theta_{Control} = \theta_{EFSL} = \theta_{ASF}$,
$H_1^{1.1} : \theta_{Control} \leq \theta_{EFSL} \leq \theta_{ASF}$ (at least one strict inequality),
where $\theta_g, g \in \{Control, ASF, EFSL\}$, is the location parameter for group g, and $F(x - \theta_g)$ is a CDF of the knowledge score Y_{ij} in group g (after rank alignment by block).
Decision rule: If the P-value from Jonckheere-Terpstra JT test statistic (Jonckheere 1954, computed using Hodges and Lehmann's (1962) aligned rank test statistic) is greater than 0.10 go to Step 5; otherwise reject $H_0^{1.1}$ and perform the next step.

Step 2 $H_0^{1.2} : \theta_{ASF} \leq \theta_{Control}$,
$H_1^{1.2} : \theta_{ASF} > \theta_{Control}$.
Decision rule: If the P-value from a Hodges and Lehmann's (1962) aligned rank test statistic is greater than 0.10 go to Step 5; if the P-value is at most 0.10 reject the null and perform the next step.

Step 3 $H_0^{1.3} : \theta_{EFSL} \leq \theta_{Control}$,
$H_1^{1.3} : \theta_{EFSL} > \theta_{Control}$.
Decision rule: If the P-value from a Hodges and Lehmann's (1962) aligned rank test statistic is greater than 0.10 go to Step 5; if the P-value is at most 0.10 reject the null and perform the next step.

Step 4 $H_0^{1.4} : \theta_{ASF} \leq \theta_{EFSL}$,
$H_1^{1.4} : \theta_{ASF} > \theta_{EFSL}$.
Decision rule: If the P-value from a Hodges and Lehmann's (1962) aligned rank test statistic is greater than 0.10 go to Step 5; if the P-value is at most 0.10 reject the null and perform the next step.

Step 5 If a sufficient number (by the analysts' judgement) responded N to $q6$, repeat Steps 1–4 but stratified by the respondent's yes and no answers to $q6$, starting with the yes responses Y_j^y. At each step replace "go to Step 5" with "stop".[1]

Estimates

We assume SUTVA and a constant additive model of effects (e.g. $Y_j(1) = Y_j(0) + \tau$) for each of the two treatments (ASF and EFSL). We use Hodges and Lehmann's (1962) aligned rank test statistic to construct 90% confidence intervals using 0.1 increments

[1]Because we are stratifying by a variable observed after intervention, we may consider this principal stratification. Given small smaple size, we keep things simple and assume that these changes were predetermined. Indeed, that seems a safe assumption as we expect most changes, if any, to be brought about by elections. If set $Z_n = \emptyset$, or very small, then ignore this step.

in τ and Hodges-Lehmann point estimates (Hodges and Lehmann 1963). If relevant, estimates and confidence intervals will be reported stratified responses by $q6$.

Discussion

This analytical plan may yield a number of different conclusions, while controlling the Type-I error rate in this set of hypotheses. For example, the available evidence may fail to reject the null of no effect from either treatment. Alternatively, it may reject that null but fail to reject the null of no effect from the strongest hypothesized treatment (ASF), thus suggesting a lack of power. If we conclude that the strongest treatment has an effect, we proceed to test the null of no effect from the hypothesized weaker treatment (EFSL). If we find that too has an effect, then we can test whether the effect of the ASF is indeed larger than that of the EFSL. If a sufficient number (by the analysts' judgement) responded no to $q6$, repeat analysis stratified by the yes no answer to that questions. We expect effects amongst those that answer yes and no effects amongst those than answered no. This would be indicative of short lived effects of treatment on awareness of treatment status.

A.1.2 Hypotheses about short-run beliefs about probability of being audited

In this section we ask how being audited for the first time changes subjective beliefs about the probability of being audited. For illustration, suppose the formation of these beliefs follows a simple (reduced-form) equation:[2]

$$\text{Prob}(A_{mt} = 1 | D_{mt}, A_{mt-1}) = F(\alpha D_{mt} - \beta A_{mt-1}),$$

where A_{mt} is an indicator variable equal to 1 if municipality m is audited in year t and zero otherwise; $D_{mt} \in [0, 1]$ captures beliefs about the sampling weight of municipality m in the audit selection process; and F is a CDF like the logit. Although we do not observe D_{mt}, we assume $D_{mt} \geq D_{m't}$ whenever municipality m has been audited more frequently than m' in recent memory. Intuitively, variable D_{mt} captures equilibrium beliefs, whereas A_{mt-1} captures dynamic adjustments in beliefs. Thus, we expect exposure to treatment to increase D_{mt} (these municipalities had never previously been audited); reduce their belief about the probability of being audited in the year immediately after the audit ($A_{mt-1} = 1$); and increase it the year after ($A_{mt-1} = 0$). This simple dynamic structure is based on our priors about how experimental subjects might form their beliefs, not on the sampling process

[2] This is for illustration. A more structured approach would include the process of bayesian learning from the past history of audits, etc.

used by the auditors. The latter may or may not include independent sampling across time.

Testing these hypothesized effects can be done directly or indirectly. The direct approach involves postulating a parametric structural model, estimating its parameters, and testing various restrictions (e.g. whether $\beta = 0$ in the previous model against the one-sided alternative that it is less than zero). In the presence of clustering, heterogeneity, and state dependence, estimating such models requires large samples and many assumptions (Greene 2008, Chp. 23). By contrast, the indirect approach is to test whether the observed outcome data can reject restrictions on a non-parametric formulation of that model. It separates the problem of estimation from that of inference. For example, for model $\text{Prob}(A_{mt} = 1|D_{mt}, A_{mt-1}) = f(D_{mt}, A_{mt-1})$ we could test $\frac{\partial f(D_{mt}, A_{mt-1})}{\partial A_{mt-1}} = 0$ against the one-sided alternative that it is less than 0. We can do this by examining responses to questions q7-q8 of the Audit Module. Q7 asks what is the subject's belief that his municipality will be audited in the current year, that is a year after the intervention. Q8 asks what is the subject's belief that his municipality will be audited next year, that is two years after the intervention. If the non-parametric model is correct, we would expect our experimental manipulation to be successful in yielding a greater number of positive difference in responses $r_{q8ijd} - r_{q7ijd}$, by individual i, in municipality j, amongst the treated ($d = 1$) relative to the controls ($d = 0$). If we interpret positive differences as a success of the intervention, then we can tabulate the successes and failures across experimental arms to test one-sided differences in success probabilities.[3]

Outcome Concepts

Subjective probabilities of being audited in the shot-run.

Measurement Instrument

Audit Module, Municipal Administration Survey (see appendix B).

Outcome Measures

$S_{ig} = \mathbf{1}(r_{q8ig} - r_{q7ig} > 0)$ $g \in \{\{EFS, ASF\}, Control\}$ measures the success in manipulating short-run belief about the probability of being audited for individual i in experimental group g, and $\mathbf{1}(.)$ is an indicator function. Note we are pooling both treatments.

Analytical Plan

[3] Directly comparing absolute differences in responses across arms violates assumptions made in almost all non-parametric tests, including symmetrical distributions. Remember, the outcome variable – the percent chance of being audited in a given year – is bounded in 0-100. Because the term D_{mt} is also likely to be much lower amongst controls, both experimental groups will likely differ, not only in the location parameter, but also in the scale and skewness parameters of the CDF of their responses, thus violating standard assumptions.

We will test the short-run dynamic effect of treatment on beliefs about the probability of being audited, using Barnard's exact test (a more powerful version of Fisher's exact test). To improve power of this test we pool all treated units into a single group, ignoring the distinction between EFSL and ASF.

Step 1 $H_0^{2.1}: \theta_1 = \theta_2 = \ldots = \theta_K = 1,$
$H_1^{2.1}: \theta_1 = \theta_2 = \ldots = \theta_K > 1,$
where the alternative suggests that the odds of success amongst the treated is larger comprated to control.
Decision rule: If the P-value from a Cochran-Mantel-Haenszel test statistic (Mantel and Haenszel 1959) with a continuity correction is greater than α, do not reject the null. Otherwise reject the null.

Estimates

As a summary measure of the strength of association we use the Maentel-Haenszel common odds ratio point estimator and confidence interval.

Discussion

Rejecting $H_0^{2.1}$ provides evidence in favor of a short-run effect of being audited, in the direction of reducing the belief that municipalities will be audited in consecutive years. That is, it provides evidence of some time dependence.

A.1.3 Hypotheses about long-run beliefs about probability of being audited

In addition to time dependence, being audited for the first time may also change the equilibrium beliefs about the probability of being audited in the future, which we try to tease out using questions q9-q11 of the Audit Module. These are derived from Manski's (2004) method of measuring expectations. Questions q9-q11 are designed to measure the binomial probability mass function $f(k; n, p)$, where k is the number of successes (audits), p the probability of success, and n the number of trials, which we set to 3 because the length of a municipal government term is 3 years and there can be only one audit per year. Q9 asks the probability of exactly one success in 3 years; q10 asks the probability of exactly two successes in the same period, and q11 asks the probability of exactly three successes. The probability of no audit in three years is defined as $f(0; 3, p) = 1 - \sum_{q \in \{q9-q11\}} r_{qij}$, with $f(0; 3, p) = 0$ if $\sum_{q \in \{q9-q11\}} r_{qij} > 1$. Finally, from the binomial distribution we note that the probability of exactly k successes in $n = 3$ trials is given by $\Pr(K = k) = \binom{n}{k} p^k (1-p)^{n-k}$. The survey responses give us the left hand side of the previous equation as reported by each subject. Because we also know n we can solve for p at each level of $k \in \{1, 2, 3\}$ given that $p \in [0, 1]$.

Outcome Concepts

Long-run subjective probabilities of being audited.

Measurement Instrument

Audit Module, Municipal Administration Survey (see appendix B).

Outcome Measures

$\bar{\sigma}_{im} = \frac{1}{3}\sum_{k=9}^{11} \min_\sigma[r_{qkij} - f(k-8;3,\sigma)] \quad s.t.\ \sigma \in [0,1]$ where $f(k-8;3,\sigma)$ is the binomial probability of $k-8$ successes in 3 independent trials with probability σ. Substantively $\bar{\sigma}_{im}$ is the estimated implicit probability of being audited as perceived by respondent i in municipality m.

Analytical Plan

We test the effect on the long-run beliefs about the probability of being audited. Because the design is block randomized, we will use the aligned rank transformation (Hodges and Lehmann 1962). After this transformation the design may be viewed as a simple one-way layout with three levels. We expect audits by the federal auditors (ASF) to have a bigger effect than audits by state-level auditors (EFSL).

Step 1 $H_0^{3.1}: \theta_{Control} = \theta_{EFSL} = \theta_{ASF}$,
$H_1^{3.1}: \theta_i \geq \theta_{Control},\ i \in \{ASF, EFSL\}$, (at least one strict inequality),
where $\theta_i,\ i \in \{Control, ASF, EFSL\}$, is the location parameter for group i, and $F(x-\theta_i)$ is a CDF of experimental subject's beliefs about the long-run probability of audit $\bar{\sigma}_{im}$ (after rank alignment).
Decision rule: If the P-value from Fligner-Wolfe FW test statistic (Hollander and Wolfe 1999, § 6.4, computed after rank alignment) is greater than α stop; otherwise reject the null and continue to next step.

Step 2 $H_0^{3.2}: \theta_g \leq \theta_{Control},\ g \in \{ASF, EFSL\}$,
$H_1^{3.2}: \theta_g > \theta_{Control}\ g \in \{ASF, EFSL\}$.
Decision rule: For $g \in \{ASF, EFSL\}$, if the P-value from a Hodges and Lehmann's (1962) aligned rank test statistic is greater than α do not reject the null; if the P-value is at most α reject the null. If both nulls are rejected perform the next step, otherwise stop.

Step 3 $H_0^{3.3}: \theta_{ASF} = \theta_{EFSL}$,
$H_1^{3.3}: \theta_{ASF} \neq \theta_{EFSL}$.
Decision rule: If the P-value from a Hodges and Lehmann's (1962) aligned rank test statistic is greater than $\alpha/2$ do not reject the null; if the P-value is at most 0.10 reject the null.

Estimates

We assume SUTVA and a constant additive model of effects (e.g. $Y_j(1) = Y_j(0) + \tau$) for each of the two treatments (ASF and EFSL). We use Hodges and Lehmann's (1962) aligned rank test statistic to construct 90% confidence intervals using 0.1 increments in τ and Hodges-Lehmann point estimates (Hodges and Lehmann 1963). If relevant, stratified responses by $q6$ will be reported.

Discussion

This analytical plan may yield a number of different conclusions. For example, the available evidence may fail to reject the null of no effect from either treatment against the alternative that at least one of them increases baseline beliefs. Alternatively, if we conclude that at least one treatment is better than control (in terms of inducing higher beliefs), then we can test whether both or only one treatment is better than control. If both treatments are effective we can test whether they have identical effects. Due to potential baseline differences (state auditors typically audit more municipalities in their state than federal ones) we cannot be sure which belief will be highest amongst the treated, so we rely on general alternatives.

A.1.4 Hypotheses about knowledge acquisition

We are interested in three related hypotheses: Does the national program of audits (whether implemented by ASF or EFSL) have a positive ordered effect on the relevant municipal administrators' knowledge of FISM grant rules and regulations? If so, do both ASF and EFSL have a positive effect or only one of them? Finally, if both are effective, how much do their effects differ if at all?

Outcome Concept

Average knowledge of FISM grant rules and regulations by relevant municipal administrators.

Measurement Instrument

Knowledge Module, Municipal Administration Survey (see appendix B).

Outcome Measures

Municipal administrators' FISM knowledge scale (Y_{im}): $Y_{im} = \sum_{q=1}^{24} r_{qim}$ is the sum score of binary responses r to questions q by municipal administrator $i \in I = \{$Municipal President, Treasurer, Director of Public Services, Director of Public Works, and/or Director of Urban Planning$\}$, in municipality m. If the answer is correct $r_{qim} = 1$, and if the answer is incorrect or the subject does not know $r_{qim} = 0$. The range of Y_{im} is

$[0-24]$ and higher scores indicate more knowledge.

Analytical Plan

Step 1 $H_0^{4.1}: \theta_{Control} = \theta_{EFSL} = \theta_{ASF}$,
$H_1^{4.1}: \theta_{Control} \leq \theta_{EFSL} \leq \theta_{ASF}$ (at least one strict inequality),
where $\theta_g,\ g \in \{Control, ASF, EFSL\}$, is the location parameter for group g, and $F(x-\theta_g)$ is a CDF of the knowledge score Y_{im} in group g after rank alignment by block.
Decision rule: If the P-value from Jonckheere-Terpstra JT test statistic (Jonckheere 1954, computed using Hodges and Lehmann's (1962) aligned rank test statistic) is greater than α stop; otherwise reject $H_0^{4.1}$ and perform the next step.

Step 2 $H_0^{4.2}: \theta_{ASF} \leq \theta_{Control}$,
$H_1^{4.2}: \theta_{ASF} > \theta_{Control}$.
Decision rule: If the P-value from a Hodges and Lehmann's (1962) aligned rank test statistic is greater than α stop; if the P-value is at most α reject the null and perform the next step.

Step 3 $H_0^{4.3}: \theta_{EFSL} \leq \theta_{Control}$,
$H_1^{4.3}: \theta_{EFSL} > \theta_{Control}$.
Decision rule: If the P-value from a Hodges and Lehmann's (1962) aligned rank test statistic is greater than α stop; if the P-value is at most α reject the null and perform the next step.

Step 4 $H_0^{4.4}: \theta_{ASF} \leq \theta_{EFSL}$,
$H_1^{4.4}: \theta_{ASF} > \theta_{EFSL}$.
Decision rule: If the P-value from a Hodges and Lehmann's (1962) aligned rank test statistic is greater than α stop; if the P-value is at most α reject the null.

Estimates

We assume SUTVA and a constant additive model of effects (e.g. $Y_j(1) = Y_j(0) + \tau$) for each of the two treatments (ASF and EFSL). We use Hodges and Lehmann's (1962) aligned rank test statistic to construct 90% confidence intervals using 0.1 increments in τ and Hodges-Lehmann point estimates (Hodges and Lehmann 1963).

Discussion

This analytical plan may yield a number of different conclusions, while controlling the Type-I error rate in this set of hypotheses.[4] For example, the available evidence

[4]Because these are sequentially partitioned hypotheses the probability of at least one Type I error in this set of hypothesis is at most α. See Rosenbaum (2009) for details.

may fail to reject the null of no effect from either treatment. Alternatively, it may reject that null but fail to reject the null of no effect from the strongest hypothesized treatment (ASF), thus suggesting a lack of power. If we conclude that the strongest treatment has an effect, we proceed to test the null of no effect from the hypothesized weaker treatment (EFSL). If we find that too has an effect, then we can test whether the effect of the ASF is indeed larger than that of the EFSL.

A.1.5 Hypotheses about FISM priorities

How does the fact of being audited impact municipal administrator's beliefs about what ought to be the priorities of the FISM? Audits may induce changes in priorities through various channels: The deterrent effect of being included in the audit sampling frame; behavioural nudges from actually being audited; or simply increased knowledge of municipal needs as a result of the audit. We divide priorities into programmatic and geographic. To test for changes in programmatic priorities we asked municipal administrators to fully allocate a budget between three types of goods: Public goods, club gods, and private transfers. To test for changes in geographic priorities we asked them to allocate a budget between the council seat and other localities in the municipality. These two outcomes are likely correlated, but not perfectly so.

Outcome Concept

Programatic and geographic spending priorities for FISM grant funds.

Measurement Instrument

Priorities Module, Municipal Administration Survey (see appendix B).

Outcome Measures

Proportion of FISM that ought to be invested outside council seat ($r_{ij}^{q1b} \in [0, 100]$), as reported by municipal administrator $i \in$ {Municipal President, Treasurer, Director of Public Services, Director of Public Works, and/or Director of Urban Planning}, in municipality j.[5] Higher responses indicate a greater proportion of investments should go to localities other than the municipal council seat.

Proportion of FISM that ought to be invested in public goods (a), club goods (b) and private goods(c) such that $r_{ij}^{q2v} \in [0, 100], v \in [a, b, c], \sum_v r_{ij}^{q2v} = 100$.

[5] In the endline survey we tried to interview the Municipal President, Treasurer, Director of Public Services, Director of Public Works, and/or Director of Urban Planning. If not available we interviewed the official immediately below in the hierarchy and so on. We do not go all the way down to an official's secretary.

Coherent overall response $Y_{ij} = \frac{1}{2}\left(r_{ij}^{q1b} + r_{ij}^{q2a}\right)$ captures how successful the audit was in improving both the geographic and programmatic allocation preferences of municipal respondents. Higher scores imply an allocation more consonant with the objectives of the FIMS grant.

Analytical Plan

To better understand this plan note that there are two dimensions of coherence in the analysis: Do both treatments have an effect, and are they effective in changing both programmatic and geographic preferences. As before we begin by pooling the treatments while focusing on the most important coherent response. If the null is rejected, the analysis bifurcates: On the one hand we test the disaggregated effects of treatments; on the other hand we test their aggregated effect on a secondary response, and so on.

Step 1 $H_0^{5.1} : \theta_{Control} = \theta_{EFSL} = \theta_{ASF}$,
$H_1^{5.1} : \theta_{Control} \leq \theta_{EFSL} \leq \theta_{ASF}$ (at least one strict inequality),
where $\theta_g, g \in \{Control, ASF, EFSL\}$, is the location parameter for group g, and $F(x - \theta_g)$ is a CDF of coherent response Y_{ij} in group g (after rank alignment by block).
Decision rule: If the P-value from Jonckheere-Terpstra JT test statistic (Jonckheere 1954, computed using Hodges and Lehmann's (1962) aligned rank test statistic) is greater than α stop; otherwise reject $H_0^{5.1}$ and perform the next step.[6]

Step 2 $H_0^{5.2} : \theta_{ASF} \leq \theta_{Control}$,
$H_1^{5.2} : \theta_{ASF} > \theta_{Control}$.
Decision rule: If the P-value from a Hodges and Lehmann's (1962) aligned rank test statistic is greater than α jump to Step 5; if the P-value is at most α reject the null and perform the next step.

Step 3 $H_0^{5.3} : \theta_{EFSL} \leq \theta_{Control}$,
$H_1^{5.3} : \theta_{EFSL} > \theta_{Control}$.
Decision rule: If the P-value from a Hodges and Lehmann's (1962) aligned rank test statistic is greater than α jump to Step 5; if the P-value is at most α reject the null and perform the next step.

Step 4 $H_0^{5.4} : \theta_{ASF} \leq \theta_{EFSL}$,

[6] The motivation for this is as follows. Subjects are asked to allocate a budget between three items. Due to the budget constraint there are only two degrees of freedom. Once they provide r_{ij}^{q2a} and r_{ij}^{q2b}, then $r_{ij}^{q2c} = 100 - r_{ij}^{q2a} - r_{ij}^{q2b}$. We are mostly interested in whether treatment increases r_{ij}^{q2a}.

$H_1^{5.4}: \theta_{ASF} > \theta_{EFSL}$.

Decision rule: If the P-value from a Hodges and Lehmann's (1962) aligned rank test statistic is greater than α jump to Step 5; if the P-value is at most α reject the null and perform the next step.

Step 5 $H_0^{5.5}: \theta_{Control} = \theta_{EFSL} = \theta_{ASF}$,
$H_1^{5.5}: \theta_{Control} \leq \theta_{EFSL} \leq \theta_{ASF}$ (at least one strict inequality),
where $\theta_g, g \in \{Control, ASF, EFSL\}$, is the location parameter for group g, and $F(x - \theta_g)$ is a CDF of response r_{ij}^{q1b} in group g (after rank alignment by block).

Decision rule: If the P-value from Jonckheere-Terpstra JT test statistic (Jonckheere 1954, computed using Hodges and Lehmann's (1962) aligned rank test statistic) is greater than α do not reject the nul and perform next step; otherwise reject $H_0^{5.1}$ and perform the next step.

Step 6 $H_0^{5.6}: \theta_{Control} = \theta_{EFSL} = \theta_{ASF}$,
$H_1^{5.6}: \theta_{Control} \leq \theta_{EFSL} \leq \theta_{ASF}$ (at least one strict inequality),
where $\theta_g, g \in \{Control, ASF, EFSL\}$, is the location parameter for group g, and $F(x - \theta_g)$ is a CDF of response r_{ij}^{q2a} in group g (after rank alignment by block).

Decision rule: If the P-value from Jonckheere-Terpstra JT test statistic (Jonckheere 1954, computed using Hodges and Lehmann's (1962) aligned rank test statistic) is greater than α stop; otherwise reject $H_0^{5.1}$ and perform the next step.

Step 7 $H_0^{5.7}: \theta_{Control} = \theta_{EFSL} = \theta_{ASF}$,
$H_1^{5.7}: \theta_{Control} \geq \theta_{EFSL} \geq \theta_{ASF}$ (at least one strict inequality),
where $\theta_g,\ g \in \{Control, ASF, EFSL\}$, is the location parameter for group g, and $F(x - \theta_g)$ is a CDF of response r_{ij}^{q2b} in group g (after rank alignment).

Decision rule: If the P-value from Jonckheere-Terpstra JT test statistic (Jonckheere 1954, computed using Hodges and Lehmann's (1962) aligned rank test statistic) is greater than α do not reject the null and perform to next step; otherwise reject $H_0^{5.1}$ and perform the next step.

Step 8 $H_0^{5.8}: \theta_{Control} = \theta_{EFSL} = \theta_{ASF}$,
$H_1^{5.8}: \theta_{Control} \geq \theta_{EFSL} \geq \theta_{ASF}$ (at least one strict inequality),
where $\theta_g,\ g \in \{Control, ASF, EFSL\}$, is the location parameter for group g, and $F(x - \theta_g)$ is a CDF of response r_{ij}^{q2c} in group g (after rank alignment by block).

Decision rule: If the P-value from Jonckheere-Terpstra JT test statistic (Jonckheere 1954, computed using Hodges and Lehmann's (1962) aligned rank test statistic) is greater than α do not reject the null; otherwise reject $H_0^{5.1}$ and perform the next step.

Estimates

We assume SUTVA and a constant additive model of effects (e.g. $Y_{ij}(1) = Y_{ij}(0) + \tau$) for each of the two treatments (ASF and EFSL) and responses $Y_{ij}, r_{ij}^{q1b}, r_{ij}^{q2a}, r_{ij}^{q2b}, r_{ij}^{q2c}$. We use Hodges and Lehmann's (1962) aligned rank test statistic to construct 90% confidence intervals using 0.1 increments in τ and Hodges-Lehmann point estimates (Hodges and Lehmann 1963).

Discussion

This analytical plan may yield a number of different conclusions, while controlling the Type-I error rate in this set of hypotheses. First, we may fail to reject the null that the combined treatments had no effect on the coherent outcome. Second, if that null is rejected then in Steps 2-4 we test for different effects of treatment by ASF and EFSL. We may conclude that we do not have power to distinguish any of these effects, or that only the ASF is effective, or both and, if so, reject the null that the effect of the stronger hypothesized treatment (ASF) is no better than the weaker one (EFSL). Third, if the null is rejected in Step 1, we also test whether the combined treatment has an effect on one or both elements of the coherent response. And if the combined treatment has a positive effect on r^{q2a}, then we test whether this increase came at the expense of one or both of the other two categories $\left(r_{ij}^{q2b}, r_{ij}^{q2c}\right)$.

A.1.6 Hypotheses about perception of municipal capacity

How do audits impact municipal administrator's perceptions of municipal capacity and their desire to engage in capacity building via training programs. A common complaint of auditors is lack of local capacity to oversee grant administration, resulting in many breaches of grant procedures. First, lack of capacity may result from changes in administration every three years and the turnover of most personnel. This is a popular explanation, though a significant proportion of new personnel may have had previous administrative experience.[7] Second, lack of capacity may be a deliberate choice whenever politicians face little incentives to invest in capacity. Third, lack of capacity may simply reflect ignorance on the part of local

[7] In the endline survey we collect individual level data from municipal administrator regarding previous administrative experience and future prospects in other levels of the administration.

administrators: They might not know what is expected of them.

A program of audits may change incentives for capacity investments through a number of channels. First, the deterrent effect may induce capacity investments to avoid harassment from auditors. This may involve training current personnel as well as hiring more qualified personnel in the future. Second, being audited may provide new information about performance expectations from federal authorities. Indeed, auditors see themselves mostly as training local administrative officials and setting a new performance bar. Finally, audits may be completely ineffective in changing perceptions, future plans to invest in capacity, and hence local administrative capacity.

Outcome Concept

Perceptions of municipal capacity and plans to improve that capacity.

Measurement Instrument

Capacity Module, Municipal Administration Survey (see appendix B).

Outcome Measures

Municipal administrators' perception of municipal capacity scale (Y_{ij}): $Y_{ij} = \sum_{k=1}^{4} r_{qkij}$ is the sum score of responses r, to questions qk, $k\ in\{1,2,3,4\}$, in measurement instrument Q, by municipal administrator $i \in$ {Municipal President, Treasurer, Director of Public Services, Director of Public Works, and/or Director of Urban Planning}, in municipality j, in the experimental group J. Responses are on a five point Likert scale where least agreement with the statement scores 5 and maximal agreement scores 0. Higher scores indicate less confidence in the municipality's capacity. We expect the audit to decrease this confidence in most cases, given widespread lack of capacity.

Municipal administrator's experience and perceptions about training (Y'_{ij}): $Y'_{ij} = \sum_{k=5}^{6} r_{qkij}$ is the sum score of responses r, to questions qk, $k\ in\{5,6\}$, in measurement instrument Q, by municipal administrator $i \in$ { Municipal President, Treasurer, Director of Public Services, Director of Public Works, and/or Director of Urban Planning}, in municipality j, in the experimental group J. There are four valid responses to the question "Have you received training in 2012?": Yes, no, plan to, don't know. The responses are ordinal, such that yes answers score a 2; plan to score 1; and no or don't know score 0. Higher scores indicate more planned or actual investments in capacity.

Coherent overall response $\bar{Y}_{ij} = \frac{1}{2}\left(Y_{ij} + Y'_{ij}\right)$: captures how successful the audit was in generating awareness about local lack of capacity relative to program goals, and the need for greater investment in training. Higher scores imply a greater effect in the predicted direction.

Analytical Plan

To better understand this plan note that there are two dimensions of coherence in the analysis: Do both treatments have an effect, and are they effective in changing both perceptions of local capacity and plans for capacity training. As before we begin by pooling the treatments while focusing on the most important coherent response. If the null is rejected, the analysis bifurcates: On the one hand we test the disaggregated effects of treatments; on the other hand we test their aggregated effect on a secondary response, and so on.

Step 1 $H_0^{6.1}: \theta_{Control} = \theta_{EFSL} = \theta_{ASF}$,
$H_1^{6.1}: \theta_{Control} \leq \theta_{EFSL} \leq \theta_{ASF}$ (at least one strict inequality),
where $\theta_g,\ g \in \{Control, ASF, EFSL\}$, is the location parameter for group g, and $F(x - \theta_g)$ is a CDF of coherent response $\bar{Y}_{ij}$ in group g (after rank alignment by block).
Decision rule: If the P-value from Jonckheere-Terpstra JT test statistic (Jonckheere 1954, computed using Hodges and Lehmann's (1962) aligned rank test statistic) is greater than α stop; otherwise reject $H_0^{6.1}$ and perform the next step.[8]

Step 2 $H_0^{6.2}: \theta_{ASF} \leq \theta_{Control}$,
$H_1^{6.2}: \theta_{ASF} > \theta_{Control}$.
Decision rule: If the P-value from a Hodges and Lehmann's (1962) aligned rank test statistic is greater than α jump to Step 5; if the P-value is at most α reject the null and perform the next step.

Step 3 $H_0^{6.3}: \theta_{EFSL} \leq \theta_{Control}$,
$H_1^{6.3}: \theta_{EFSL} > \theta_{Control}$.
Decision rule: If the P-value from a Hodges and Lehmann's (1962) aligned rank test statistic is greater than α jump to Step 5; if the P-value is at most α reject the null and perform the next step.

Step 4 $H_0^{6.4}: \theta_{ASF} \leq \theta_{EFSL}$,
$H_1^{6.4}: \theta_{ASF} > \theta_{EFSL}$.
Decision rule: If the P-value from a Hodges and Lehmann's (1962) aligned rank test statistic is greater than α jump to Step 5; if the P-value is at most α reject the null and perform the next step.

[8] The motivation for this is as follows. Subjects are asked to allocate a budget between three items. Due to the budget constraint there are only two degrees of freedom. Once they provide r_{ij}^{q2a} and r_{ij}^{q2b}, then $r_{ij}^{q2c} = 100 - r_{ij}^{q2a} - r_{ij}^{q2b}$. We are mostly interested in whether treatment increases r_{ij}^{q2a}.

Step 5 Repeat the above steps with Y_{ij} and Y'_{ij} as the responses.

Estimates

We assume SUTVA and a constant additive model of effects (e.g. $Y_{ij}(1) = Y_{ij}(0) + \tau$) for each of the two treatments (ASF and EFSL) and responses $\bar{Y}_{ij}, Y_{ij}, Y'_{ij}$. We use Hodges and Lehmann's (1962) aligned rank test statistic to construct 90% confidence intervals using 0.1 increments in τ and Hodges-Lehmann point estimates (Hodges and Lehmann 1963).

Discussion

This analytical plan may yield a number of different conclusions, while controlling the Type-I error rate in this set of hypotheses. For example, the available evidence may fail to reject the null of no effect from either treatment. Alternatively, it may reject that null but fail to reject the null of no effect from the strongest hypothesized treatment (ASF), thus suggesting a lack of power. If we conclude that the strongest treatment has an effect, we proceed to test the null of no effect from the hypothesized weaker treatment (EFSL). If we find that too has an effect, then we can test whether the effect of the ASF is indeed larger than that of the EFSL. Finally, if we reject the null of no effect on the coherent response, we proceed to analyze effect son each of the two components of the response, as above.

A.1.7 Hypotheses about compliance with FISM reporting and data accessibility rules

Do audits lead to improvements in municipal compliance with FISM reporting and data accessibility requirements? We focus on these aspects of compliance because, unlike citizen participation, they are relatively easy to measure.[9]

Outcome Concept

Compliance with FISM reporting and data accessibility rules

Measurement Instrument

Compliance survey (see Appendix B).

Outcome Measures

Municipalities compliance success score (Y_j): $Y_j = \sum_{k=1}^{4} r_{qkj}$, is the sum score of re-

[9]Other requirements would include informing SEDESOL, involving citizens at the planning and evaluation stage through COMPLADEMUNS, doing performance evaluations, and so on. Many of these cannot be measured without doing another endline audit.

sponses r, to items $qk, k \in \{1, \ldots, 4\}$, as recorded by a research assistant. In particular, $r_{q1j} = 1$ if the municipality has a web page, and 0 otherwise; $r_{q2j} = 1$ if the Plan de Desarrollo Municipal is available through the web, telephone or email inquiry, and 0 otherwise; $r_{q3j} = 1$ if four or more reports are available, and 0 otherwise; $r_{q4j} = 1$ if the Plan de Inversiones FISM is available through the web, telephone or email inquiry, and 0 otherwise. Municipalities that succeed in all four aspects score a 4, those that fail all items score 0.

Analytical Plan

Step 1 $H_0^{7.1} : \beta = 0$,
$H_1^{7.1} : \beta > 0$.
where β is a parameter of the local log odds ratio (see Agresti, Mehta and Patel (1990) for details). Specifically, $\beta = 0$ implies independence between the row and column variables. $\beta > 0$ implies a positive monotone relation. The row variable captures the ordinal treatment arm (control=1, EFSL=2, and ASF=3) and the ordinal column variable captures the five response values (0 through 4). The three way table has dimensions $3 \times 5 \times 17$.
Decision rule: If the P-value from a conditional (e.g. stratified) linear-by-linear test statistic (Agresti, Mehta and Patel 1990) is greater than α stop; otherwise reject $H_0^{7.1}$.[10]

Estimates

With such a small sample it might not be possible to provide precise estimates. MLE estimates will be provided by fitting an ordered logit model with a latent single index of the form $y_j^* = \beta_1 d_j + \beta_2 s_j + \varepsilon_j$, where y_j is the ordered column outcome variable, d_j is the ordered row treatment variable, and s_j is the blocking factor.

Discussion

Rejecting the null will provide evidence that the national program of audits had some effect in improving compliance with program rules.

A.1.8 Hypotheses about the actual allocation of FISM grants

Do audits help improve the geographic allocation of FISM grants between council seat and outlying localities, and the programmatic allocation across public, club, and private goods?

Outcome Concept

[10]The conditional linear by linear test is implemented in the Coin package for R software.

Geographic and programmatic allocation of investments in compliance with FISM priorites

Measurement Instrument

Quarterly municipal reported data to federal treasury (SHCP) via the online reporting tool known as *Portal Aplicativo de la Secretaría de Hacienda* (PASH) on the use of FISM investment funds.

Outcome Measures

Proportion of FISM investments going to municipal council seat (Y_{tj}): Using the last quarterly report for 2012 let $Y_{yj} = \frac{1}{M_{yj}} \sum_{i=1}^{M_{yj}} r_{yij}$, where M_{yj} is the number of investments made in municipality j in year $y = 2012$, and where $r_{yij} = 1$ if the investment is located outside the municipal council seat and zero otherwise.[11]

Proportion of FISM investments going to public goods (Y'_{tj}): We categorize all types of investments into three types: Public goods, club goods, and private goods, according to a classification algorithm. $Y'_{tj} = \frac{1}{\sum_{i=1}^{M_{yj}} V_{iyj}} \sum_{i=1}^{M_{yj}} \mathbf{1}(V_{iyj})V_{iyj}$, where M_{yj} is the number of investments made in municipality j in year $y = 2012$, $\mathbf{1}(.)$ is an indicator function equal to 1 whenever expenditures V_{iyj} fall in the public goods category and 0 otherwise.

Coherent response (Y''_{tj}): The coherent response $Y''_{tj} = \frac{1}{2}\left(Y_{tj} + Y'_{tj}\right)$ captures how successful the audit is in improving the self-reported geographic and programmatic allocation of investments in the year after the audit.

Analytical Plan

Unlike other outcomes in this study, we do have several years of pre-treatment outcomes from previous reports that can be used as covariates for covariance adjustments. These covariates can help improve the precision of our inferences, as described in Rosenbaum (2002*a*). Specifically, we use the fixed effects predictive model $X_{t,j} = \alpha_j + \beta_1 X_{t-1,j} + \beta_2 X_{t-2,j} + \varepsilon_{t,j}$ to predict outcomes $\widehat{X}_{t,j}$, for $X_{t,j} = \text{logit}(Z_{t,j})$, $Z_{t,j} \in \{Y_{tj}, Y'_{tj}, Y''_{tj}\}$. Inference is carried out using the covariance adjusted cross-section of outcomes $e_{tj} = X_{t,j} - \widehat{X}_{t,j}$, as in Rosenbaum (2002*a*).

To better understand this plan note that there are two dimensions of coherence in the analysis: Do both treatments have an effect, and are they effective in changing both programmatic and geographic preferences. As before we begin by pooling the treatments while focusing on the most important coherent response. If the null is rejected, the analysis bifurcates: On the one hand we test the disaggregated effects of treatments; on the other hand we test their aggregated effect on a secondary response, and so on.

[11] Our outcome variable is for the year as a whole but based on the last quarterly report, which is typically the most reliable report as it includes all updates and revisions during the year (communication with ASF).

Step 1 $H_0^{8.1}: \theta_{Control} = \theta_{EFSL} = \theta_{ASF}$,
$H_1^{8.1}: \theta_{Control} \leq \theta_{EFSL} \leq \theta_{ASF}$ (at least one strict inequality),
where $\theta_i,\ i \in \{Control, ASF, EFSL\}$, is the location parameter for group i, and $F(x - \theta_i)$ is a CDF of the covariance adjusted coherent response e''_{ij}.
Decision rule: If the P-value from Jonckheere-Terpstra JT test statistic (Jonckheere 1954, computed using Wilcoxon's (1945) rank sum test statistic) is greater than α stop; otherwise reject $H_0^{8.1}$ and perform the next step.

Step 2 $H_0^{8.2}: \theta_{ASF} \leq \theta_{Control}$,
$H_1^{8.2}: \theta_{ASF} > \theta_{Control}$.
Decision rule: If the P-value from a Wilcoxon's (1945) rank sum test statistic is greater than α jump to Step 5; if the P-value is at most α reject the null and perform the next step.

Step 3 $H_0^{8.3}: \theta_{EFSL} \leq \theta_{Control}$,
$H_1^{8.3}: \theta_{EFSL} > \theta_{Control}$.
Decision rule: If the P-value from a Wilcoxon's (1945) rank sum test statistic is greater than α jump to Step 5; if the P-value is at most α reject the null and perform the next step.

Step 4 $H_0^{8.4}: \theta_{ASF} \leq \theta_{EFSL}$,
$H_1^{8.4}: \theta_{ASF} > \theta_{EFSL}$.
Decision rule: If the P-value from a Wilcoxon's (1945) rank sum test statistic is greater than α jump to Step 5; if the P-value is at most α reject the null and perform the next step.

Step 5 $H_0^{8.5}: \theta_{Control} = \theta_{EFSL} = \theta_{ASF}$,
$H_1^{8.5}: \theta_{Control} \leq \theta_{EFSL} \leq \theta_{ASF}$ (at least one strict inequality),
where $\theta_i,\ i \in \{Control, ASF, EFSL\}$, is the location parameter for group i, and $F(x - \theta_i)$ is a CDF of the covariance adjusted response e_{ij}.
Decision rule: If the P-value from Jonckheere-Terpstra JT test statistic (Jonckheere 1954, computed using Wilcoxon's (1945) rank sum test statistic) is greater than α do not reject the null and perform next step; otherwise reject $H_0^{8.1}$ and perform the next step.

Step 6 $H_0^{8.6}: \theta_{Control} = \theta_{EFSL} = \theta_{ASF}$,
$H_1^{8.6}: \theta_{Control} \leq \theta_{EFSL} \leq \theta_{ASF}$ (at least one strict inequality),
where $\theta_i,\ i \in \{Control, ASF, EFSL\}$, is the location parameter for group i, and $F(x - \theta_i)$ is a CDF of the covariance adjusted response e'_{ij}.
Decision rule: If the P-value from Jonckheere-Terpstra JT test statistic (Jon-

ckheere 1954, computed using Wilcoxon's (1945) rank sum test statistic) is greater than α stop; otherwise reject $H_0^{8.1}$ and perform the next step.

Estimates

We assume SUTVA and a constant additive model of effects (e.g. $X_{t,j}(1) = X_{t,j}(0) + \tau$, where $X_{t,j}$ is the logit transformation of the relevant outcome) for each of the two treatments (ASF and EFSL) and the three outcomes $Y_{ij}, Y'_{ij}, Y''_{ij}$. We use Wilcoxon's (1945) rank sum test statistic to construct 90% confidence intervals using small increments in τ and Hodges-Lehmann point estimates (Hodges and Lehmann 1963).

Discussion

This analytical plan may yield a number of different conclusions, while controlling the Type-I error rate in this set of hypotheses. First, we may fail to reject the null that the combined treatments had no effect on the coherent outcome. Second, if that null is rejected then in Steps 2-4 we test for different effects of treatment by ASF and EFSL. We may conclude that we do not have power to distinguish any of these effects, or that only the ASF is effective, or both and, if so, reject the null that the effect of the stronger hypothesized treatment (ASF) is no better than the weaker one (EFSL). Third, if the null is rejected in Step 1, we also test whether the combined treatment has an effect on one or both elements of the coherent response.

A.2 Secondary objective: Differences between state and federal audits

In this section we compare the effectiveness with which the federal and state level auditors uncover wrongdoings; the severity with which they judge them; and the diligence with which they pursue wrongdoings. If solid evidence of differences is found, we can do some additional exploratory work to be tested in future experiments. For example, any such differences could be driven by differences in audit durations, staffing, quality of personnel, incentives, institutional independence, and so on.

A.2.1 Hypotheses about effectiveness

Does the ASF yield a greater number of observations? Does the ASF recover grater transfer amounts for the federal treasury? Are effects moderated by the party affinity of the governor and municipality being audited? Or by the independence score of the relevant auditor?

We expect the federal auditors (ASF) to be more independent, unbiased and technically competent that the average state-level auditors (EFSL). First, suppose municipality j has a sample I_j of FISM expenditure items audited, $I_j = \{i_1, i_2, \ldots, i_{n_j}\}$. Let $\Xi = \{\xi_1, \xi_2, \ldots, \xi_l\}$ be the set of numbered admissibility criteria used to judge each FISM expenditure item $i_j \in I_j$. Let the total number of observations in municipality j be defined by $\theta_j = \sum_{k=1}^{n_j} \mathbf{1}(i_k \notin \Xi; ASF_j)$, where $\mathbf{1}(.; ASF_j)$ is an indicator function equal to 1 if item i_j does not fall into any of the l admissible categories in Ξ as interpreted by the relevant auditor ($ASF_j =$ 1 or $ASF_j = 0$) and zero otherwise. In English, the total number of observations θ_j in municipality j is a mapping from the set of audited items I_j, the criteria Ξ used to judge the elements in this set of items, and whether the audit is performed by the ASF ($ASF_j = 1$) or an EFSL ($ASF_j = 0$). The main hypothesis is that $\frac{\partial \theta_j}{\partial ASF_j} > 0$. The precise behavioural rationale (beyond this mechanical exposition) can be found in the accompanying theoretical paper.

Second, by a similar logic we expect the ASF to be able to recover more misspent funds. Some of the observations discussed previously lead to requests for funds to be returned to the federal treasury. Of the refunds, some are recorded by the auditors as actual (i.e. refunded *in situ*), and others as probable (i.e. pending refund). The refund total is the sum of these two components. Presumably a more feared/respected auditor will get more actual refunds. A weak EFSL might report the same total but fail to actually deliver the funds, as it could always blame municipal intransigence for the lack of follow up. The randomized intervention will allow us to peer past this veil. In expectation actual refunds ought to be the same across auditors.

Finally, testing these hypotheses can be confounded by party affinity between local majors and state governors (W, see previous section) and the degree of independence of the relevant auditor, as measured by Figueroa Neri (2009). For example, EFSL may under-report observations in municipalities affine with the state governor, and over-report observations for those in opposition. The former may inflate, and the former deflate, the main effect of being audited by the ASF, with the overall tendency a function of the distribution of W. Because we did not stratify by party affinity, this binary variable may take the same or different values across municipalities audited by ASF and EFSL in the same state. Formally, let $\theta_j = \sum_{k=1}^{n_j} \mathbf{1}(i_k \notin \Xi; ASF_j, W_j)$ determine the number of observations θ_j. We assume $\frac{\partial \theta_{ij}}{\partial W} = 0$ when $ASF_j = 1$, or that municipal party affinity has no impact on municipalities audited by ASF. This allows a simplification. With municipalities paired by states, with one audited by the ASF the other by EFSL, there are four possible values for the vector

(W_{isd}, W_{jsd}).[12] The assumption says that the only relevant value in this pair is the one associated with the municipality audited by the EFSL. This allows us to limit the heterogeneity by collapsing the 4×2 factorial "experiment" (in quotes because the levels of the first factor were not randomized) into a 2×2 experiment, which is helpful in a very small sample like ours.

In theory we could further distinguish this effect by stratifying by the index of auditor independence. Thus, we might expect party affinity to only matter in cases where the EFSL is not very independent, say. Such finessing is hard to accomplish in a small sample like ours. Even if we collapse the index into three categories, and concern ourselves only with the value of party affinity in municipalities audited by the EFSL, that still provides 3×2 possible moderator levels, for a 6×2 factorial experiment. Hence, we will not test the moderation effects in combination, but do separate test for party affinity and auditor independence.

Outcome Concept

Relative independence, unbiasedness and technical competence of superior audit institutions.

Measurement Instrument

Institutional survey (see Appendix B).

Outcome Measures

Number of observations per municipal FISM audit (Y_{js}): $Y_{js} = \theta_{js}$ (see discussion in previous section).

Amount of federal refunds (Y'_{js}): $Y'_{js} = R_{js}$, where R_{js} is the total amount of refunds reported by the auditors for municipality j in state s.

Coherent outcome (Y^*): $Y^* = \frac{1}{2}(Rank(Y_{js}) + Rank(Y'_{js}))$, where $Rank(x)$ is a function that ranks the full set of municipalities in the experimental group from lowest to highest on the level of $x, x \in \{Y_{js}, Y'_{js}\}$. This gives approximately equal weight to standardized outcomes (Rosenbaum 2009, pg. 300).

State and municipal governments party affinity (W_{js2012}): $W_{js2012} = 1$ if the ruling party (singly or in coalition) in state s also forms part of the municipal government (singly or in coalition) in municipality j on the first day of year 2012 and municipality j was audited by an EFSL. $W_{js2012} = 0$ otherwise.[13]

[12] E.g. (0,0), (0,1), (1,0), (1,1).

[13] Because we are looking at budgeted expenditures, we assume these are formed in the expectation that the incumbent at the start of the year remains in power. It might be the case that the municipal government is initially of the same party but is expected to loose an upcoming election, in which case the governor

Auditor independence index (H_s): Where H_s is an index in the range 0-100 of the degree of independence of the audit institutions in state s, or of the federal auditor for $s = 0$. For details of the index constructions see Figueroa Neri (2009).
Coarsened independence index ($\widehat{H}_s$): Where $\widehat{H}_s = 1$ if the score of municipality s is in the bottom third in the most recent year for which the index is available; $\widehat{H}_s = 2$ if the score of municipality s is in the middle third; and $\widehat{H}_s = 3$ if the score of municipality s is in the top third.

Analytical Plan

Step 1 $H_0^{9.1} : \theta_{ASF} = \theta_{EFSL}$,
$H_1^{9.1} : \theta_{ASF} > \theta_{EFSL}$
where $\theta_i,\ i \in \{ASF, EFSL\}$, is the location parameter for group i, and $F(x - \theta_i)$ is a CDF of the coherent outcome Y^* in group i.[14]
Decision rule: If the P-value from a Wilcoxon's (1945) signed rank statistic is greater than α go to Step 4; otherwise reject $H_0^{9.1}$ and perform the next step.

Step 2 $H_0^{9.2} : \theta_{ASF} = \theta_{EFSL}$,
$H_1^{9.2} : \theta_{ASF} > \theta_{EFSL}$
where $\theta_i,\ i \in \{ASF, EFSL\}$, is the location parameter for group i, and $F(x - \theta_i)$ is the number of observations per municipal audit Y in group i.
Decision rule: If the P-value from a Wilcoxon's (1945) signed rank statistic is greater than α do not reject the null and continue to next step; otherwise reject $H_0^{1.1}$ and perform the next step.

Step 3 $H_0^{9.3} : \theta_{ASF} = \theta_{EFSL}$,
$H_1^{9.3} : \theta_{ASF} > \theta_{EFSL}$
where $\theta_i,\ i \in \{ASF, EFSL\}$, is the location parameter for group i, and $F(x - \theta_i)$ is the number of observations per municipal audit Y'_{js} in group i.
Decision rule: If the P-value from a Wilcoxon's (1945) signed rank statistic is greater than α do not reject the null; otherwise reject $H_0^{9.1}$.

Step 4 $H_0^{9.4} : (\theta_{ASF,W=1} - \theta_{EFSL,W=1}) \leq (\theta_{ASF,W=0} - \theta_{EFSL,W=0})$,
$H_1^{9.4} : (\theta_{ASF,W=1} - \theta_{EFSL,W=1}) > (\theta_{ASF,W=0} - \theta_{EFSL,W=0})$,
where $\theta_i,\ i \in \{ASF, EFSL\}$, is the location parameter for group i, and $F(x - \theta_i)$ is a CDF of the coherent outcome Y^* in group i.
Decision rule: If Patel and Hoel's (1973) T statistic is such that $\frac{\nu^{1/2}T}{\hat{\sigma}} \geq z_{1-\alpha}$

might not favor it. However, randomization ensures these conditions will balance out in expectations across treatment arms.

[14] As we are comparing matched pairs, no need for rank alignment. They are equivalent.

reject the null and proceed to next step. Otherwise do not reject the null and stop.

Step 5 $H_0^{9.5}: (\theta_{ASF,W=1} - \theta_{EFSL,W=1}) \leq (\theta_{ASF,W=0} - \theta_{EFSL,W=0})$,
$H_1^{9.5}: (\theta_{ASF,W=1} - \theta_{EFSL,W=1}) > (\theta_{ASF,W=0} - \theta_{EFSL,W=0})$,
where $\theta_i,\ i \in \{ASF, EFSL\}$, is the location parameter for group i, and $F(x - \theta_i)$ is a CDF of the number of observations per municipal audit Y in group i.
Decision rule: If Patel and Hoel's (1973) T statistic is such that $\frac{\nu^{1/2}T}{\hat{\sigma}} \geq z_{1-\alpha}$ reject the null and proceed to next step. Otherwise do not reject the null and proceed to next step.

Step 6 $H_0^{9.6}: (\theta_{ASF,W=1} - \theta_{EFSL,W=1}) \leq (\theta_{ASF,W=0} - \theta_{EFSL,W=0})$,
$H_1^{9.6}: (\theta_{ASF,W=1} - \theta_{EFSL,W=1}) > (\theta_{ASF,W=0} - \theta_{EFSL,W=0})$,
where $\theta_i,\ i \in \{ASF, EFSL\}$, is the location parameter for group i, and $F(x - \theta_i)$ is a CDF of the number of observations per municipal audit Y in group i.
Decision rule: If Patel and Hoel's (1973) T statistic is such that $\frac{\nu^{1/2}T}{\hat{\sigma}} \geq z_{1-\alpha}$ reject the null and proceed to next step. Otherwise do not reject the null and proceed to next step.

Step 7 We repeat the precious three steps but stratifying by $\widehat{H}$, and adjusting for the fact that the latter has three levels.

Estimates

We assume SUTVA and a constant additive model of effects (e.g. $Y_j(1) = Y_j(0) + \tau$) for each treatment effect hypothesized above (even if it was not tested). We use Hodges and Lehmann's (1962) aligned rank test statistic to construct 90% confidence intervals using 0.1 increments in τ and Hodges-Lehmann point estimates (Hodges and Lehmann 1963) for all effects hypothesized above (even if it was not tested).

Discussion

Once again this analytical plan can provide a number of insights. Given that ASF and EFSL audit comparable municipalities (in expectation), failure to reject the coherent null would suggests no main effect from being audited by the ASF versus EFSL. However, this does not rule out the possibility of confounding if heterogeneous effects cancel out across levels of a moderator. That is, we need to test for offsetting interactions before we can conclude that the National Program of Audits is homogeneous across ASF and EFSL. Rejection of the coherent null suggests ASF audits yield more observations or result in more refund claims, which we proceed to test separately. Failure to reject either subsidiary test likely reflects a loss of power from the more disaggregated outcomes.

We have hypothesized two possible sources of heterogeneous effects: local party affinity (W) and the independence of state level auditors (H). Due to small sample size we test them separately for interaction effects with the treatment. Because moderators were not randomized we do not test for their main effects. If we reject the null of no interaction effect with treatment in the determination of the coherent outcome, then we test for effects on the components of that outcome.

A.2.2 Hypotheses about severity

First, auditors' observations are classified into 11 ordered categories, from least bad to worse (ASF 2009, pg. 33-35). For example, the least severe observation is a "Recommendation" and the highest "Fine". Auditors also issue an overall opinion about the audited entity. Opinions fall into four ordered categories: Clean opinion, qualified opinion, negative opinion, and abstention of opinion (ASF 2009, pg. 33-35). The latter happens when a municipality could not be audited or it did not provide the documentation necessary for the audit (unit non-response). Does the ASF issue more severe observations, harsher opinions? Second, auditor's also issue different reasons for reimbursements. Specifically, reimbursements are sorted into pre-specified reasons, which we treat as unordered categories. Does the ASF provide systematically different reasons for its reimbursement decisions relative to EFSL?

Outcome Concept

Severity of auditor and auditor diagnostic

Measurement Instrument

Institutional survey (see appendix **??**) and the Results Report for the Audit of the 2010 Public Accounts.[15]

Outcome Measures

Average municipal observation score (Y_{js}): Y_{js} is calculated by scoring observation categories from 1 to 11, in order of least to most serious, assigning the relevant score to each and every observation in a municipality j, and averaging them. For $O = \{o_1, o_2, \ldots, o_i, \ldots, o_n\}$ observations in municipality j, with corresponding scores $S = \{s_1, s_2, \ldots, s_i, \ldots, s_n\}$, the average municipal score in municipality j is $Y_s = \frac{1}{n}\sum_{i=1}^{n} s_i$

Overall auditor opinion of municipal handling of FISM transfers (Y'_{js}): Y'_{js} is an ordered

[15]The report is available at http://www.asf.gob.mx under the title *Informe del Resultado de la Fiscalizacin Superior de la Cuenta Pública 2010.*

categorical variable with four levels as described in the previous section. However, one of those levels is "abstention from opinion". Instead of dropping these municipalities, we will give them an average score of 2. The final measure has levels 1 through 3.
Auditor diagnostic ($\mathbf{A}_g$): $= \sum_j \mathbf{A}_{gj}$ where $\mathbf{A}_{gj} = (x_1, x_2, \ldots, x_i, \ldots, x_{11})^T$ is an 11×1 column vector whose elements x_i count the number of observations falling under nominal diagnostic category i in the audit report for municipality j according to auditor $g \in \{ASF, EFSL\}$.
State and municipal governments party affinity (W_{js2012}): See Hypothesis A.2.1.
Coarsened independence index ($\widehat{H}_s$): See Hypothesis A.2.1.

Analytical Plan

Step 1 $H_0^{10.1} : \theta_{ASF} = \theta_{EFSL}$,
$H_1^{10.1} : \theta_{ASF} > \theta_{EFSL}$
where $\theta_i,\ i \in \{ASF, EFSL\}$, is the location parameter for group i, and $F(x - \theta_i)$ is a CDF of the average municipal observation score Y in group i.
Decision rule: If the P-value from a Wilcoxon's (1945) signed rank statistic is greater than α go to the next step; otherwise reject $H_0^{10.1}$ and perform the next step.

Step 2 $H_0^{10.2} : (\theta_{ASF,W=1} - \theta_{EFSL,W=1}) \leq (\theta_{ASF,W=0} - \theta_{EFSL,W=0})$,
$H_1^{10.2} : (\theta_{ASF,W=1} - \theta_{EFSL,W=1}) > (\theta_{ASF,W=0} - \theta_{EFSL,W=0})$,
where $\theta_i,\ i \in \{ASF, EFSL\}$, is the location parameter for group i, and $F(x - \theta_i)$ is a CDF of the average municipal observation score Y in group i.
Decision rule: If Patel and Hoel's (1973) T statistic is such that $\frac{\nu^{1/2}T}{\hat{\sigma}} \geq z_{1-\alpha}$ reject the null and proceed to next step. Otherwise do not reject the null and stop.

Step 3 We repeat the previous step but stratifying by $\widehat{H}$, and adjusting for the fact that the latter has three levels. Whether the null is rejected or not proceed to next step.

Step 4 $H_0^{10.4} : \mathbf{A}_{ASF} \overset{d}{=} \mathbf{A}_{EFSL}$,
$H_1^{10.4} : \mathbf{A}_{ASF} \overset{d}{\neq} \mathbf{A}_{EFSL}$.
this is a standard chi-square test of homogeneity. Since auditors are randomized to municipalities the distribution of observations falling under the different diagnostic criteria ought to be the same in expectation across the two groups of audited municipalities. The alternative is that the distributions are not homogeneous presumably because both auditors are not identical at diagnosing.

Decision rule: If the P-value from a permuted χ^2 statistic is less than α reject the null, otherwise do not reject and stop.

Step 5 Repeat previous step but using the overall auditor opinion of municipal handling of FISM transfers (Y'_{js}) as the outcome.

Estimates

We assume SUTVA and a constant additive model of effects (e.g. $Y_j(1) = Y_j(0) + \tau$) for each treatment effect hypothesized in Steps 1-3 above (even if it was not tested). We use Wilcoxon's signed rank test statistic and Patel and Hoel's (1973) T statistic as appropriate to construct 90% confidence intervals using 0.1 increments in τ and Hodges-Lehmann point estimates (Hodges and Lehmann 1963) for all effects hypothesized above (even if it was not tested).

Discussion

Once again this analytical plan can provide a number of insights. Given that

A.3 Tertiary objective: Interactions with local accountability systems

In Mexico the constitutional ban on consecutive terms severely handicaps individual-level electoral accountability. How then are incumbents held to account? And who holds them to account if not voters: Political parties, other career related principals, or accountability agencies like the ASF or the judiciary? We posit three channels of accountability. First, incumbent officials may be held to account by political parties. In this scenario voters hold parties accountable through the re-election incentive, and parties hold incumbents accountable by screening candidates or rewarding good candidates with political careers. Beyond political parties, candidates may have career concerns in the private sector so they will be accountable to the interests of future employers. A term in office provides a very visible opportunity for incumbents to invest in their reputation and thereby improve their future career prospects.[16] Unlike parties, that need to please voters to be re-elected, private sector principals may not have interests in common with the voters. Third, incumbents may be held to account by accountability agencies such as the ASF. However, because the ASF has limited powers to punish dissonant behaviour in office, it has to rely on public opinion, political parties, or other institutions like the judiciary to deal out punishments and rewards and hold incumbents to account.

[16] For a sense of Mexican political career paths see Diaz-Cayeros (2006, §3 and 4).

As with private sector principals the ASF may not have the same objectives as the majority of voters in a given municipality. Consequently, the effect, if any, of an auditors' opinion on incumbent career prospects is unclear. Essentially, principals holding incumbents to account may interpret the evidence released by the the audit differently. For example, incumbents may bend program rules to satisfy a certain constituency. Bending the rules may score badly with the auditors but well with the beneficiary constituency. Indeed, confirmation by the auditors that the incumbent has been favouring a specific constituency may improve the incumbents career prospect with that constituency. (It may also worsen their career prospects with constituencies, hitherto unaware of such favouritism, that are hurt by it.) Matters are more straightforward when the criteria of auditors and principals coincide. However, because we do not know whom the principals are in any given context we will limit ourselves for the most part to two-sided hypotheses.

We randomize audits, not audit reports. Audited municipalities receive an overall opinion from the auditors that we code into three levels: High, Medium, and Low. Municipalities with a High score are those that have performed best according to the auditors evaluation. In principle this score is a deterministic function of the behaviour of the municipal administration in the year *prior* to the audit. That is, the score is a covariate defining three strata as shown in Table 1. For controls we only observe the marginal distribution of outcomes $\mathrm{P}(Y_C)$, while for treated units we observe the marginal and conditional distribution $\mathrm{P}(Y_T) = \sum_{i \in \{H,M,L\}} \mathrm{P}(Y_T|i)\mathrm{P}(i)$.

In general the effect of the audit report depends on whether principals discriminate on the basis of performance in office, whether they sort performance perfectly, and whether their evaluation is aligned with that of the ASF (e.g. by rewarding those getting high marks from ASF). First, if principals do not know the distribution of types $\mathrm{P}(i)$ $i \in \{H, M, L\}$, they may lump all incumbents together as average. Second, principals may know the marginal distribution of types $\mathrm{P}(i)$ but not be able to perfectly sort incumbents into the right bin. That is, they may divide incumbents into two (or more) groups in the right proportion but who goes into which group has some error. If so average outcomes in Table 1 would be the same across experimental conditions, assuming we could observe outcomes under Control. Put differently, the experiment may affect individual outcomes (some people are shuffled around) but not average outcomes (the shuffling cancels out). Third, the ASF's scores may be positively or negatively aligned with the principals' evaluation metric, or not aligned at all in which case the ASF scores are ignored.

With the above possibilities in mind we can say that a necessary but not sufficient condition

**Incumbent behaviour** ($t-1$)	**Principals' assessment** (t)							
	Control				Treatment			
	Low	Med.	High	Total	Low	Med.	High	Total
Good	1	3	5	9	-	-	9	9
Average	2	5	2	9	-	9	-	9
Bad	4	4	1	9	9	-	-	9
Total	7	12	8	27	9	9	9	27
Payoff	-1	0	1	-	-1	0	1	-
Avg. outcome	-	-	-	$\bar{Y}_C$	$\bar{Y}_T^L$	$\bar{Y}_T^M$	$\bar{Y}_T^H$	$\bar{Y}_T$

Table 1: Example of how principals may classify 27 incumbents on the basis of their behaviour on the year prior to the audit (an audit at time t audits accounts of $t-1$). The first column is the hypothesized behaviour of the incumbent in the year prior to the audit which is only known with certainty by the incumbent. As given in the column labeled "Total" 9 incumbents had good behaviour, 9 bad and 9 average. Without good information on this behaviour principals in this example classify too many incumbents as average, and too few as good or bad as can be seen along the row labelled "Total" (in fact principals may only be able to classify incumbents on a coarser measure, like a binary Good/Bad classification). By contrast we assume auditors do observe the true incumbent behaviour at $t-1$, report it truthfully and, in this example, induce perfect sorting by principals. Auditor errors in forming an opinion, deliberate misreporting, or failure by principals to fully trust auditor reports would show up in the off-diagonal elements in the Treatment panel. The penultimate row describes some possible payoffs to being perceived as Low, Medium or High. The important assumption here is that payoffs are at least weakly increasing (or decreasing) in type. The last row shows the outcomes actually observed by the researchers (e.g. incumbent responses to survey questions about career prospects and so on). For treated incumbents we can stratify responses by audit score. For controls we only observe the marginal average $\bar{Y}_C$.

for the ASF scores to have an effect is for them to be aligned.[17] Furthermore, for the effect to be detectable there must be some lumping together of incumbents in one or more bins in the Control condition.[18] Hence, one way to test for an effect is to look for an increase in the variance of outcomes across treatment and control arms. In addition we can also test the necessary but not sufficient condition of aligned ASF scores by testing whether scores help predict outcomes amongst the treated.

A.3.1 Hypotheses about future political appointments

Do municipal administrators receiving low opinions from the auditors have lower expectations of a political career compared to control? This test only makes sense if enough municipalities receive a low opinion which, judging by past audit results, about 40% of them do.

Outcome Concept

Subjects' expectations about future political appointments

Measurement Instrument

Career Concerns Module, Municipal Administration Survey (see appendix B).

Outcome Measures

Subjects expectations about future political appointments (Y_{ij}): $Y_{ij} = 1$ if the response $r_{q7ij} \in \{1, 2, 3\}$ and zero otherwise. That is, the variable is coded as 1 whenever the subject selects any one of the first three options, from amongst the 5 options available, as a response to Question 7. The first three options indicate an expectation of a future political appointment.

Analytical Plan

Step 1 $H_0^{1.1} : p_T^1 = p_C^1,\ p_T^2 = p_C^2,\ \ldots,\ p_T^K = p_C^K$;
$H_1^{1.1} : p_T^k \leq p_C^k\ \forall k$ (with at least one inequality strict);
where p_T^k is the probability that $Y_{ij} = 1$ in block k and so on. Here we only include in the treatment group municipalities receiving a low opinion from the auditor, discarding all other treated municipalities. The null is that the probability of success p is orthogonal to treatment status, conditional on the block factors. (We pool treated units to include those audited by either ASF or

[17]Not sufficient because even if aligned they may provide no new information.

[18]If the marginal marginal distribution of types $\mathrm{P}(i)$ are the same across treatment and control and treatment only shifts incumbents across bins, then some incumbents benefit and others loose from treatment with little or no effect on average across bins.

EFSL.) The alternative is that subjects in the treatment group have systematically lower probabilities of success.

Decision rule: If the p-value from Mantel and Haenszel's (1959) MH statistic is greater than α do not reject the null and proceed to next step. Otherwise reject the null and proceed to next step. (These are not nested hypotheses.)

Step 2 As above but comparing responses of municipal administrators in municipalities with high auditor opinions to control and taking care to reverse the inequality in $H_1^{1.1}$.

Estimates

The Mantel and Haenszel (1959) statistic test the significance of an association between treatment status and the probability of expecting a political career. However, it does not measure the degree of association. An interesting estimate is the differential log odds of success across treatment and control conditions. We estimate a logit model and then compute the plugging estimator as detailed in Freedman (2008). The linear predictor used for estimation is $Y_{ij} = \beta_0 + \beta_1 B_{ij} + \beta_2 H_{ij} + \beta_2 M_{ij} + \beta_2 L_{ij} + \varepsilon_{ij}$, where B_{ij} is a block dummy, H_{ij} is a dummy taking a value of 1 when the auditor's opinion for municipality j is High and 0 otherwise, and so on for the Medium (M) and Low (L) dummies.

Discussion

This analytical plan may yield a number of different conclusions. However, because the two hypotheses are not nested, the Type-I error rate in this set of hypotheses is underestimated. Rejection of the first hypothesis provides some evidence that auditor scores are positively aligned and low opinions by the auditors have an impact on incumbent political career prospects. It does not show that the association is causal however, as we do not also stratify controls by audit score. At most this provides some weak evidence in favor of the political party channel of accountability. However, failure to reject the null does suggest bad audit reports have no discernible effects on incumbent career prospects, either because audit scores are irrelevant or because they are redundant (e.g. principals are already sorting perfectly). The second hypothesis tests accountability for positive outcomes. It might be that bad news are discounted, and parties only react to good performers.

A.3.2 Hypotheses about career prospects

How do audits impact municipal administrator's perceptions of career prospects?

Outcome Concept

Subjects expectations of future career prospects.

Measurement Instrument

Career Concerns Module, Municipal Administration Survey (see appendix B).

Outcome Measures

Subjects' expectations about future career prospects (Y'_{ij}): $Y_{ij} \sum_{k=9}^{11} r_{qkij}$, is the sum score of responses r, to questions $qk, k \in \{9, 10, 11\}$, of the Career Concerns survey module. Each response is based on a five point Likert scale, with least agreement scoring 1 and most agreement scoring 5. For analysis we treat the response as if it were continuous.

Analytical Plan

Step 1 $H_0^{2.1} : F\left(\frac{Y_T - \theta_T}{\sigma_T}\right) = F\left(\frac{Y_C - \theta_C}{\sigma_C}\right) \quad -\infty < t < \infty$,
$H_1^{2.1} : \theta_T \neq \theta_C$ and/or $\sigma_T \neq \sigma_C$
where $\theta_i, i \in \{\{ASF, EFSL\}, Control\}$, is the location parameter for group i, σ_i is its scale parameter, and $F\left(\frac{Y_i - \theta_i}{\sigma_i}\right)$ is a CDF of subjects' expectations about future career prospects (Y'_{ij}) (after rank alignment). Under the alternative both groups have the same distribution but with different parameters (a more general but less informative test is a Kolmogorov-Smirnov test).
Decision rule: If the P-value from a Lepage (1971) two-sample location-scale statistic (after rank alignment) is greater than α stop; otherwise reject $H_0^{2.2}$ and perform the next step.

Step 2 $H_0^{2.2} : \gamma^2 = 1$,
$H_1^{2.2} : \gamma^2 > 1$
where $\gamma^2 \equiv \frac{\sigma_T^2}{\sigma_C^2}$.
Decision rule: If the P-value from a permuted $T(Z, y) = \gamma^2$ statistic comparing the ratio of the variances amongst treated and control groups across all possible treatment assignments is greater than α go to the next step; otherwise reject the null and perform the next step.

Step 3 $H_0^{2.3} : \theta_T^L = \theta_T^M = \theta_T^H$,
$H_1^{2.3}$: At least one inequality,
where $\theta_T^j, j \in \{\text{Low, Medium, High}\}$, is the location parameter for treated units in stratum j and $F(x - \theta_T^j)$ is a CDF of subjects' expectations about future career prospects (Y'_{ij}) (after rank alignment).
Decision rule: If the P-value from Kruskal-Wallis test statistic (computed using Hodges and Lehmann's (1962) aligned rank test statistic) is greater than α stop;

otherwise reject $H_0^{2.2}$ and perform the next step.

Step 4 $H_0^{2.4} : \theta_T^j = \theta_T^i$,

$H_1^{2.4} : \theta_T^j \neq \theta_T^i$ for $i \neq j$, $i, j \in$ {Low, Medium, High}.

Decision rule: If the Steel-Dwass-Critchlow-Fligner two-sided all-treatments multiple comparison statistic $|W_{ij}| \geq w_\alpha^*$ decide $\theta_T^j \neq \theta_T^i$ and stop; otherwise decide $\theta_i = \theta_j$. (See Hollander and Wolfe (1999, pgs. 240–244) for implementation details.)

Estimates

First we provide a point estimate and confidence interval for γ as described in Hollander and Wolfe (1999, § 5). Next, we assume SUTVA and normally though not identically distributed disturbances (i.e. we allow for heteroscedasticity) and estimate the following model for treatment outcomes using robust OLS: $Y_T = \beta_1 + \beta_2 \text{High} + \beta_3 \text{Low}$ where the inputs High and Low are dummy variables equal to 1 if the unit receives a high or low score from the ASF respectively.

Discussion

This analytical plan may yield a number of different conclusions, while controlling the Type-I error rate in this set of hypotheses. Rejection of the first null provides evidence that treatment has shifted the location and/or the scale of the marginal distribution of outcomes across experimental arms. The second null tests whether the marginal distributions have different variances, if so then we have some evidence that the treatment is improving discrimination by principals as perceived by incumbents. Next we test whether the ASF scores are aligned (though if the null in Stpe 2 was not rejected they may be redundant, as noted above). First by checking whether there is any difference between outcomes across any strata (principals may only be using a two bin classification scheme thus lumping two strata together) and, second, by testing which strata are different. We can look at the estimates to gauge whether the alignment is positive or negative.

A.3.3 Hypotheses about municipal administrators' rank ordering of principals

Outcome Concept

Subjects rank ordering of the ASF amongst possible principals.

Measurement Instrument

Career Concerns Module, Municipal Administration Survey (see appendix B).

Outcome Measures

Subjects rank ordering of ASF (Y^*_{ij}): $Y^*_{ij} = 2$ if municipal administrators include the ASF amongst the top three principals they are most concerned about in response to Question 8 (a). $Y^*_{ij} = 3$ if municipal administrators list ASF as topmost amongst the three most important in response to Question 8 (b). $Y^*_{ij} = 1$ otherwise. We expect the treated to be more likely to report a higher score.

Analytical Plan

Step 1 $H_0^{3.1} : p_1 = p_2 = p_3$, where $p_j = Pr(D = 1|Y = j, B = k)$, $j = 1, 2, 3$, $B = 1, \ldots, 17$ is the block, and $D = 1$ if unit is assigned to treatment by either ASF or EFSL, and zero otherwise.

$H_1^{3.1} : p_1 \leq p_2 \leq p_3$ (with at least one inequality strict).

Essentially we define 17 different 3×2 contingency tables, where the three rows are scores and the two columns control and treatment. The null is that there is no association between the tables' column and row variables. The alternative is that there is a positive association.

Decision rule: If the p-value of Mantel's (1963) extension statistic is at most α, reject the null and proceed to next step.[19] Otherwise do not reject the null and stop.

Step 2 $H_0^{3.2} : p_1 = p_2 = p_3$, where $p_j = Pr(D = 1|Y = j, B = k)$, $j = 1, 2, 3$, $B = 1, \ldots, 17$ and $D = 1$ if unit is assigned to treatment by ASF and zero otherwise (here we drop units treated by EFSL).

$H_1^{3.2} : p_1 \leq p_2 \leq p_3$ (with at least one inequality strict).

Same test as above but dropping units exposed to audit by EFLS so we contrast ASF to control only.

Decision rule: If the p-value of Mantel's (1963) extension statistic is at most α, reject the null and proceed to next step. Otherwise do not reject the null and proceed to next step.

Step 3 $H_0^{3.3} : p_1 = p_2 = p_3$, where $p_j = Pr(D = 1|Y = j, B = k)$, $j = 1, 2, 3$, $B = 1, \ldots, 17$ and $D = 1$ if unit is assigned to treatment by EFSL and zero otherwise (here we drop units treated by ASF).

$H_1^{3.3} : p_1 \leq p_2 \leq p_3$ (with at least one inequality strict).

Decision rule: If the p-value of Mantel's (1963) M^2 extension statistic is at most α, reject the null. Otherwise do not reject the null.

[19] On the use of this test see also Rosenbaum (2002*b*, pg 32).

Estimates

We fit a logit model as in Agresti (2010, §6.4).

Discussion

This analytical plan may yield a number of different conclusions, while controlling the Type-I error rate in this set of hypotheses. If the first null is rejected, there is evidence of a positive association between treatment and ranking of the auditor as principal. The next two hypotheses ask whether this is common to both levels of the treatment or just one of the levels.

A.3.4 Hypotheses about governors' reactions

By definition, control municipalities cannot be required to reimburse funds to the federal treasury. Audited municipalities, on the other hand, often have to reimburse some money to federal government. Are they compensated for these losses by greater transfers from the governor? If so, does the amount of compensation depend on the political affiliations of mayors relative to governors? Evidence of this behaviour would questions the deterrent effect of audits. Besides, since the governor faces a budget constraint, presumably the funds are coming from other municipalities that may not have done anything wrong. Stratify by political allegiance.

Outcome Concept

Compensation of auditor fines by governor's FISM transfer in the subsequent year to audited municipalities.

Measurement Instrument

Institutional survey (see Appendix B).

Outcome Measures

Log yearly change in budgeted gubernatorial FISM transfers (ΔY_{js2012}): $\Delta Y_{js2012} = Y_{js2012} - Y_{js2011}$, is the change in the natural log of budgeted FISM transfers for municipality j, in state s, between calendar years 2011 and 2012. Budgeted transfers are reported at the beginning of the year in which the budget applies. Actual transfers may differ.

State and municipal governments party affinity (W_{js2012}): $W_{js2012} = 1$ if the ruling party (singly or in coalition) in state s also forms part of the municipal government (singly or in coalition) in municipality j on the first day of year 2012. $W_{js2012} = 0$

otherwise.[20]

Log yearly change in municipal refunds of FISM fund to federal government (ΔM_{js2012}): The amount that the auditors have requested the municipality return to the federal government after the audit. These arise from expenditures outside the program rules. This amount is zero for all municipality years where no audit takes place, so ΔM_{js2012} in any given year is simply the total refund amount requested for an audited municipality and zero otherwise.

Analytical Plan

The analytical plan has three parts. First, we test the null that being audited by either ASF or EFSL has no main effect on the change in gubernatorial FISM transfers (ΔY_{js2012}). Second, if the previous null is rejected, we test the null of a homogeneous effect across levels of the party affinity variable (W_{jst}). Although W_{jst} is a post-treatment variable, we treat it as a covariate.[21] Third, if the previous null is rejected we test for an association between the amounts refunded to the federal government and the change in gubernatorial FISM transfer.

Step 1 $H_0^{4.1}: \theta_{Control} \geq \theta_{Treatment}$,
$H_1^{4.1}: \theta_{Control} < \theta_{Treatment}$
where $\theta_i,\ i \in \{Control, Treatment\}$, is the location parameter for group i, and $F(x - \theta_i)$ is a CDF of the change in gubernatorial FISM transfers (ΔY_{js2012}) in group i (after rank alignment).
Decision rule: If the P-value from a Hodges and Lehmann's (1962) aligned rank test statistic is greater than α stop; otherwise reject $H_0^{4.1}$ and perform the next step.

Step 2 $H_0^{4.2}: (\theta_{Treatment,W=1} - \theta_{Control,W=1}) \leq (\theta_{Treatment,W=0} - \theta_{Control,W=0})$,
$H_1^{4.2}: (\theta_{Treatment,W=1} - \theta_{Control,W=1}) > (\theta_{Treatment,W=0} - \theta_{Control,W=0})$.
Decision rule: After rank alignment, if Patel and Hoel's (1973) T statistic is such that $\frac{\nu^{1/2}T}{\hat{\sigma}} \geq z_{1-\alpha}$ reject the null and proceed to next step. Otherwise do

[20] Because we are looking at budgeted expenditures, we assume these are formed in the expectation that the incumbent at the start of the year remains in power. It might be the case that the municipal government is initially of the same party but is expected to loose an upcoming election, in which case the governor might not favor it. However, randomization ensures these conditions will balance out in expectations across treatment arms.

[21] The 2011 program of audits audited the 2010 accounts. The final report is not released to the general public until 2012, so it is unlikely to affect voter behaviour. However, incumbents in audited municipalities learned the results in 2011, and were expected to start making repayments from then on. Some moneys are recovered as the audit progresses in 2011, others later in 2012. Some moneys may never be recovered. Affected municipalities may call on the governor to help them finance repayments. Hence we expect treatment to affect transfers but not electoral outcomes in 2011 and possibly 2012.

not reject the null and proceed to next step.[22]

Step 3 $H_0^{4.3} : \tau = 0,$
$H_1^{4.3} : \tau > 0,$
where Kendall's τ measures the population correlation between the log yearly change in gubernatorial FISM transfers (ΔY_{js2012}) and the log yearly change in municipal refunds of FISM fund to federal government (ΔM_{js2012}).
Decision rule: Reject the null if $K \geq k_\alpha$ and proceed to next step. Otherwise do not reject the null and stop.[23]

Step 4 $H_0^{4.4} : \tau_{W=1} = 0,$
$H_1^{4.4} : \tau_{W=1} > 0,$
where Kendall's τ measures the population correlation between the log yearly change in gubernatorial FISM transfers (ΔY_{js2012}) and the log yearly change in municipal refunds of FISM fund to federal government (ΔM_{js2012}) for the subsample with $W = 1$.
Decision rule: Reject the null if $K \geq k_\alpha$ and proceed to next step. Otherwise do not reject the null and proceed to next step.

Step 5 $H_0^{4.5} : \tau_{W=0} = 0,$
$H_1^{4.5} : \tau_{W=0} > 0,$
where Kendall's τ measures the population correlation between the log yearly change in gubernatorial FISM transfers (ΔY_{js2012}) and the log yearly change in municipal refunds of FISM fund to federal government (ΔM_{js2012}) for the subsample with $W = 0$.
Decision rule: Reject the null if $K \geq k_\alpha$. Otherwise do not reject the null.

Estimates

With regards to the change in gubernatorial FISM transfers (ΔY_{js2012}), we assume SUTVA and a constant additive model of effects (e.g. $Y_j(1) = Y_j(0) + \tau$). We use Hodges and Lehmann's (1962) aligned rank test statistic to construct 90% confidence intervals using 0.005 increments in τ and Hodges-Lehmann point estimates (Hodges and Lehmann 1963). We provide estimates and confidence intervals for the sample as a whole as well as for subsamples defined by party affinity (W)

For estimates of the correlation we rely on the procedure in Hollander and Wolfe (1999, pg. 382), and for upper confidence bounds on the procedure in Hollander and Wolfe (1999, pg. 385). We provide estimates and confidence intervals for the sample as a

[22] See Keele, McConnaughy and White's (2012) online Annex for details of the procedure.
[23] See Hollander and Wolfe (1999, pgs. 363–377) for details of Kendall's testing procedure.

whole as well as for subsamples defined by party affinity (W)

Discussion

Rejecting the null will provide evidence that governors are offsetting some of the fines imposed by the auditors. Since governors face a budget constraint, these monies likely come at the expense of other current investments or redistribution of allocations across state municipalities. It raises questions as to whom ultimately pays the price for dissonant behaviour. Rejecting the null of no interaction effect provides some clues as to which municipalities benefit the most from the governor's largess. Rejecting the null of no association between fines and governor transfers, provides some evidence as to the motivation behind the causal effect.[24]

References

Agresti, A. 2010. *Analysis of Ordinal Categorical Data*. Wiley Series in Probability and Statistics Wiley.

Agresti, Alan, Cyrus R. Mehta and Nitin R. Patel. 1990. "Exact Inference for Contingency Tables with Ordered Categories." *Journal of the American Statistical Association* 85(410):453–458.

ASF. 2009. Informe del Resultado de la Revisión y Fiscalización Superior de la Cuenta Pública 2007. Technical Report I Auditoría Superior de la Federación.

Diaz-Cayeros, Alberto. 2006. *Federalism, Fiscal Authority, and Centralization in Latin America*. Cambridge University Press.

Figueroa Neri, Aimée. 2009. Buenas, malas o raras. Las leyes mexicanas de fiscalización superior (2000-2009). Technical report Auditoría Superior de la Federación.

Freedman, David A. 2008. "Randomization Does Not Justify Logistic Regression." *Statist. Sci.* 23:237–249.

Greene, William H. 2008. *Econometric Analysis*. Pearson International Edition.

Hodges, J. L., Jr. and E. L. Lehmann. 1962. "Rank Methods for Combination of Independent Experiments in Analysis of Variance." *The Annals of Mathematical Statistics* 33(2):482–497.

[24]We only test for association since we could not and did not randomize the fines, only the assignment to audit or not.

Hodges, J. L., Jr. and E. L. Lehmann. 1963. "Estimates of Location Based on Rank Tests." *The Annals of Mathematical Statistics* 34(2):598–611.

Hollander, M. and D.A. Wolfe. 1999. *Nonparametric Statistical Methods*. Wiley-Interscience.

Jonckheere, A. R. 1954. "A Distribution-Free k-Sample Test Against Ordered Alternatives." *Biometrika* 41(1/2):133–145.

Keele, Luke, Corrine McConnaughy and Ismail White. 2012. "Strengthening the Experimenters Toolbox: Statistical Estimation of Internal Validity." *American Journal of Political Science* pp. 1–16.

Lepage, Yves. 1971. "A Combination of Wilcoxon's and Ansari-Bradley's Statistics." *Biometrika* 58(1):213–217.

Manski, C.F. 2004. "Measuring expectations." *Econometrica* 72(5):1329–1376.

Mantel, N. and W. Haenszel. 1959. "Statical aspects of the analysis of data from retrospective studies of disease." *Journal of the National cancer Institute* 22(4):v.

Mantel, Nathan. 1963. "Chi-Square Tests with One Degree of Freedom; Extensions of the Mantel- Haenszel Procedure." *Journal of the American Statistical Association* 58(303):690–700.

Patel, Kantilal M. and David G. Hoel. 1973. "A Nonparametric Test for Interaction in Factorial Experiments." *Journal of the American Statistical Association* 68(343):615–620.

Rosenbaum, Paul R. 2002*a*. "Covariance Adjustment in Randomized Experiments and Observational Studies." *Statistical Science* 17:286–327.

Rosenbaum, Paul R. 2002*b*. *Observational Studies*. Springer.

Rosenbaum, P.R. 2009. *Design of observational studies*. Springer Verlag.

Wilcoxon, F. 1945. "Individual comparisons by ranking methods." *Biometrics Bulletin* 1(6):80–83.

B Survey Forms

Estimado NOMBRE DEL ENCUESTADO:

Mi nombre es NOMBRE DEL ENCUESTADOR, trabajo para la empresa Data OPM y estamos realizando un proyecto de investigación para la Universidad de Yale. El proyecto esta a cargo de la Dra. Ana De La O y tiene como objetivo entender como los profesionistas que trabajan en el gobierno municipal toman decisiones y perciben a su municipio.

Su experiencia como parte del gobierno municipal es invaluable para el proyecto. ¿Estaría usted dispuesto a completar una encuesta telefónica para este proyecto?

La encuesta toma menos de 20 minutos. Todas sus respuestas (así como su decisión de participar en la encuesta) son confidenciales. Solo los investigadores involucrados en este estudio tendrán acceso a la información que usted provea.

Su participación en este estudio es voluntaria. Usted puede dar por terminada la encuesta en cualquier momento o dejar de contestar algunas preguntas.

Para agradecer su valiosa participación en nuestro estudio, el equipo de investigación le regalara una suscripción de cuatro números a la revista Este País.

Si tiene preguntas acerca de esta encuesta, Usted puede contactar directamente a la investigadora encargada del proyecto al (52 55) 53-51-26-92 o enviar un correo electrónico a la siguiente dirección: ana.delao@yale.edu.

Si desea hablar con alguien ajeno al proyecto para compartir sus preguntas, o para discutir sus derechos como participante de esta encuesta, Usted puede ponerse al contacto con el Comité responsable de cudiar los derechos de entrevistados llamado *Human Subjects Committee* de la universidad de Yale, Box 208010, New Haven, CT 06520-8010, 203-785-4688, human.subjects@yale.edu. Información adicional esta disponible en el siguiente link: http://www.yale.edu/hrpp/participants/index.html

*QUESTION 1 *CODES 101L1
X1) Genero [1-1]
1: Masculino
2: Femenino

*QUESTION 2 *NUMBER 102L2
X2) Edad [2-3]

*QUESTION 4 *ALPHA 104L50
X3) Lugar de nacimiento   [4-53]

*QUESTION 54 *ALPHA 154L50
X4) Grado de estudios   [54-103]

*QUESTION 104 *ALPHA 204L50
X5) Empleo actual   [104-153]

*QUESTION 154 *ALPHA 254L50
X6) Area [154-203]

*QUESTION 204 *ALPHA 304L50
X7) Empleo anterior   [204-253]

*QUESTION 254 *ALPHA 354L50
X8) Correo electrónico [254-303]

*QUESTION 304 *NUMBER 404L20
X9) Teléfonos [304-323]

*QUESTION 324 *CODES 424L1
MODULO: CARRERA PROFESIONAL

P1) ¿Cada gobierno municipal tiene una duración de tres años, en cuantos gobiernos municipales ha trabajado usted a lo largo de su carrera profesional? [324-324]
1: Uno
2: Dos
3: Tres
4: Cuatro
5: Cinco o mas

*QUESTION 325 *CODES 425L1
p2) ¿Alguna vez ha formado usted parte de un gobierno estatal? [325-325]
1: Si
2: No

*QUESTION 326 *CODES 426L1
p3) ¿Alguna vez ha formado usted parte del congreso estatal? [326-326]
1: Si
2: No

*QUESTION 327 *CODES 427L1
p4) ¿Alguna vez ha formado usted parte de un gobierno federal? [327-327]
1: Si
2: No

*QUESTION 328 *CODES 428L1
p5) ¿Alguna vez ha formado usted parte del congreso federal? [328-328]
1: Si, especifique a cual________

2: No

*QUESTION 329 *ALPHA 429L100 *IF[Q328,1]
p5a) especifique a cual? [329-428]

*QUESTION 429 *CODES 529L1
p6) ¿Ocupa usted, o ha ocupado usted, algún cargo en un partido político? [429-429]
1: Si Especifique partido y cargo _________
2: No

*QUESTION 430 *ALPHA 530L100 *IF[Q429,1]
p6a) Especifique partido y cargo _________ [430-529]

*QUESTION 530 *CODES 630L1
p7) ¿Qué actividad profesional espera desempeñar una vez que termine su gestión municipal? LEER OPCIONES [530-530]
1: Trabajar en el próximo gobierno municipal
2: Trabajar en el próximo gobierno estatal
3: Trabajar en el gobierno federal
4: Negocio Propio
5: Otro Especifique____

*QUESTION 531 *ALPHA 631L50 *IF[Q530,5]
p7a) Otro Especifique____ [531-580]

*QUESTION 581 *CODES 681L1
p8a) ¿Qué actividad profesional espera desempeñar una vez que termine su gestión municipal? Elegir solamente los 3 que más le preocupan de la siguiente lista LEER OPCIONES (MENCION 1) [581-581]
1: Auditoria superior de la federación
2: Empresarios locales
3: Residentes fuera de la cabecera municipal
4: Residentes dentro de la cabecera municipal
5: Presidente Municipal
6: Sindicatos
7: Su partido u organización política
9: No contesto

*QUESTION 582 *CODES 682L1
p8b) ¿A la hora de rendir cuentas sobre su desempeño en el municipio, la opinión de quien le preocupa más? Elegir solamente los 3 que más le preocupan de la siguiente lista LEER OPCIONES (MENCION 2) [582-582]
1: Auditoria superior de la federación
2: Empresarios locales
3: Residentes fuera de la cabecera municipal
4: Residentes dentro de la cabecera municipal
5: Presidente Municipal
6: Sindicatos
7: Su partido u organización política
9: No contesto

*QUESTION 583 *CODES 683L1
p8c) ¿A la hora de rendir cuentas sobre su desempeño en el municipio, la opinión de quien le preocupa más? Elegir solamente los 3 que más le preocupan de la siguiente lista LEER OPCIONES (MENCION 3) [583-583]
1: Auditoria superior de la federación
2: Empresarios locales
3: Residentes fuera de la cabecera municipal

4: Residentes dentro de la cabecera municipal
5: Presidente Municipal
6: Sindicatos
7: Su partido u organización política
9: No contesto

*QUESTION 584 *ALPHA 684L50
p9) De estos 3, ¿Qué opinión es la que más le preocupa? ____ [584-633]

*QUESTION 634 *CODES 734L1
Una vez terminada la actual legislatura municipal, y pensando en sus perspectivas laborales, que tan de acuerdo está con los siguientes: Para cada frase dígame si está totalmente de acuerdo, algo de acuerdo, algo en desacuerdo o totalmente en desacuerdo.
p10) El hecho de haber trabajado en este ayuntamiento mejorará sus perspectivas laborales [634-634]
1: Totalmente de acuerdo
2: Algo de acuerdo
3: Algo en desacuerdo
4: Totalmente en desacuerdo
5: No sabe - No contesto

*QUESTION 635 *CODES 735L1
p11) En su siguiente ocupación, espera recibir mejor salario [635-635]
1: Totalmente de acuerdo
2: Algo de acuerdo
3: Algo en desacuerdo
4: Totalmente en desacuerdo
5: No sabe-No contesto

*QUESTION 636 *CODES 736L1
p12) En su entorno social esta bien considerado el haber sido un servidor público [636-636]
1: Totalmente de acuerdo
2: Algo de acuerdo
3: Algo en desacuerdo
4: Totalmente en desacuerdo
5: No sabe-No contesto

*QUESTION 637 *CODES 737L1
Modulo: Conocimiento del Fondo de Infraestructura Social Municipal

Ahora le voy a preguntar sobre el Fondo de Aportaciones para la Infraestructura Social Municipal (FISM)
De lo que sabe o ha oído ¿Permiten las disposiciones normativas del Fondo de Aportaciones para la Infraestructura Social Municipal (FISM) financiar los siguientes rubros?

p13) Alcantarillado [637-637]
1: Si
2: No
9: NS

*QUESTION 638 *CODES 738L1
p14) Mejoramiento de vivienda [638-638]
1: Si
2: No

9: NS

*QUESTION 639 *CODES 739L1
p15) Caminos rurales [639-639]
1: Si
2: No
9: NS

*QUESTION 640 *CODES 740L1
p16) Kioskos [640-640]
1: Si
2: No
9: NS

*QUESTION 641 *CODES 741L1
p17) Fiestas patronales [641-641]
1: Si
2: No
9: NS

*QUESTION 642 *CODES 742L1
p18) Drenaje y letrinas [642-642]
1: Si
2: No
9: NS

*QUESTION 643 *CODES 743L1
p19) Infraestructura básica de salud [643-643]
1: Si
2: No
9: NS

*QUESTION 644 *CODES 744L1
p20) Remodelación de Iglesias [644-644]
1: Si
2: No
9: NS

*QUESTION 645 *CODES 745L1
p21) Infraestructura básica de educación [645-645]
1: Si
2: No
9: NS

*QUESTION 646 *CODES 746L1
p22) Agua potable [646-646]
1: Si
2: No
9: NS

*QUESTION 647 *CODES 747L1
p23) Programas de Desarrollo Institucional [647-647]
1: Si
2: No
9: NS

*QUESTION 648 *CODES 748L1
p24) ¿Sabe usted si se puede o no gastar los fondos del FISM en obras y acciones sociales que no beneficien directamente a la población en rezago social y pobreza

extrema? [648-648]
1: Si
2: No
9: NS

*QUESTION 649 *CODES 749L1
p25) ¿Tiene su municipio la obligación de publicar, en su órgano local de difusión o página electrónica, informes trimestrales sobre el ejercicio y destino del fondo? [649-649]
1: Si
2: No
9: NS

*QUESTION 651 *CODES 751L1
p27) ¿Está su municipio obligado a informar sobre el ejercicio y destino del FISM a las siguientes instituciones: SEDESOL [651-651]
1: Si
2: No
9: NS

*QUESTION 652 *CODES 752L1
p28) ¿Está su municipio obligado a informar sobre el ejercicio y destino del FISM a las siguientes instituciones: SHCP [652-652]
1: Si
2: No
9: NS

*QUESTION 653 *CODES 753L1
p29) ¿Está su municipio obligado a informar sobre el ejercicio y destino del FISM a las siguientes instituciones: SSP [653-653]
1: Si
2: No
9: NS

*QUESTION 654 *CODES 754L1
p30) ¿Está su municipio obligado a informar sobre el ejercicio y destino del FISM a las siguientes instituciones: SEP   [654-654]
1: Si
2: No
9: NS

*QUESTION 655 *CODES 755L1
p31) ¿Está su municipio obligado a informar sobre el ejercicio y destino del FISM a las siguientes instituciones: Gobierno Estatal [655-655]
1: Si
2: No
9: NS

*QUESTION 656 *CODES 756L1
p32) ¿Está su municipio obligado a informar sobre el ejercicio y destino del FISM a las siguientes instituciones: H. Congreso de la Unión [656-656]
1: Si
2: No
9: NS

*QUESTION 657 *CODES 757L1
p33) El Sistema de Formato Único, ¿Es una herramienta de internet creada por el

gobernador estatal para mejorar la gestión del FISM? [657-657]
1: Si
2: No
9: NS

*QUESTION 658 *CODES 758L1
p34) ¿Es necesaria la participación del Comité para la Planeación del Desarrollo Municipal (COPLADEMUN) o, en su defecto, el Consejo de Desarrollo Social Municipal (CDM), para programar las obras e inversiones del fondo en su municipio? [658-658]
1: Si
2: No
9: NS

*QUESTION 659 *CODES 759L1
p35) ¿La participación de los anteriores comités es también necesaria para el seguimiento y evaluación del fondo? [659-659]
1: Si
2: No
9: NS

*QUESTION 660 *CODES 760L1
p36) ¿El gobierno del estado deposita los recursos del FISM en una cuenta bancaria en su municipio de manera anual?   [660-660]
1: Si
2: No
9: NS

*QUESTION 662 *CODES 762L1
p37) ¿Tiene el municipio que reportar a la Secretaría de Hacienda y Crédito Público (la SHCP) que áreas del municipio estuvieron encargadas del ejercicio y destino de los recursos del FISM ? [662-662]
1: Si
2: No
9: NS

*QUESTION 663 *NUMBER 763L3 *MIN 0 *MAX 100

Modulo: Prioridades acerca del FISM
Del 100% de los recursos del FISM, ¿que porcentaje considera Usted que se debe gastar en la cabecera municipal,
p39) 1. Cabecera municipal   [663-665]

*QUESTION 666 *NUMBER 766L3 *MIN 0 *MAX 100

p40)2. y que porcentaje en el resto de su municipio?   [666-668]

*QUESTION 669 *NUMBER 769L3 *MIN 0 *MAX 100
Del 100% de los recursos del FISM, ¿que porcentaje considera Usted que se debe gastar en cada uno de los siguientes tipos de servicios públicos en su municipio?

p41) Servicios como salud, clínicas, drenaje, caminos, y puentes   [669-671]

*QUESTION 672 *NUMBER 772L3 *MIN 0 *MAX 100
p42) Servicios como parques, dispensarios, plazas, kioscos y centros comunitarios [672-674]

*QUESTION 675 *NUMBER 775L3 *MIN 0 *MAX 100
p43) Servicios como mejoramiento de vivienda, desayunos escolares, y despensas

[675-677]

*QUESTION 678 *NUMBER 778L3 *MIN 0 *MAX 100
Del 100% de los recursos del FISM, ¿que porcentaje considera Usted que le gustaría a la población que se gastase en cada uno de los siguientes tipos de servicios públicos en su municipio?
p44) Servicios como salud, clínicas, drenaje, caminos, y puentes [678-680]

*QUESTION 681 *NUMBER 781L3 *MIN 0 *MAX 100
p45) Servicios como parques, dispensarios, plazas, kioscos y centros comunitarios [681-683]

*QUESTION 684 *NUMBER 784L3 *MIN 0 *MAX 100
p46) Servicios como mejoramiento de vivienda, desayunos escolares, y despensas [684-686]

*QUESTION 687 *NUMBER 787L3 *MIN 0 *MAX 100
Si el ayuntamiento de un municipio vecino al suyo quisiera ganar las elecciones locales, del 100% de los recursos del FISM, ¿que porcentaje considera Usted que se debería gastar en cada uno de los siguientes tipos servicios públicos en su municipio?

p47) Servicios como salud, clínicas, drenaje, caminos, y puentes [687-689]

*QUESTION 690 *NUMBER 790L3 *MIN 0 *MAX 100
p48) Servicios como parques, dispensarios, plazas, kioscos y centros comunitarios [690-692]

*QUESTION 693 *NUMBER 793L3 *MIN 0 *MAX 100
p49) Servicios como mejoramiento de vivienda, desayunos escolares, y despensas [693-695]

*QUESTION 696 *NUMBER 796L3 *MIN 0 *MAX 100
De los siguientes servicios públicos, ¿qué porcentaje de las necesidades cree Usted que se cubren en la Cabecera municipal?

p50) Agua potable [696-698]

*QUESTION 699 *NUMBER 799L3 *MIN 0 *MAX 100
p51) Drenaje y alcantarillado [699-701]

*QUESTION 702 *NUMBER 802L3 *MIN 0 *MAX 100
p52) Alumbrado público [702-704]

*QUESTION 705 *NUMBER 805L3 *MIN 0 *MAX 100
p53) Pavimentación [705-707]

*QUESTION 708 *NUMBER 808L3 *MIN 0 *MAX 100
De los siguientes servicios públicos, ¿qué porcentaje de las necesidades cree Usted que se cubren en el resto de su Municipio?

p54) Agua potable [708-710]

*QUESTION 711 *NUMBER 811L3 *MIN 0 *MAX 100
p55) Drenaje y alcantarillado [711-713]

*QUESTION 714 *NUMBER 814L3 *MIN 0 *MAX 100
p56) Alumbrado público [714-716]

*QUESTION 717 *NUMBER 817L3 *MIN 0 *MAX 100
p57) Pavimentación [717-719]

*QUESTION 720 *CODES 820L1
Modulo: Auditorias

p58) ¿Ha odio usted hablar de la Entidad de Fiscalización Estatal?   [720-720]
1: Si
2: No
9: NS

*QUESTION 721 *CODES 821L1
p59) ¿Ha odio usted hablar de la Auditoria superior de la federación?   [721-721]
1: Si
2: No
9: NS

*QUESTION 722 *CODES 822L1
p60) ¿Sabe usted si la ASF tiene el poder legal para revisar el manejo, por parte de los municipios, de los fondos del Ramo 33? [722-722]
1: Si
2: No
9: NS

*QUESTION 723 *CODES 823L1
p61) ¿Fue su municipio auditado el año pasado (2011) por la Entidad de Fiscalización Estatal? [723-723]
1: Si
2: No
9: NS

*QUESTION 724 *CODES 824L1
p62) ¿Fue su municipio auditado el año pasado (2011) por la Auditoria Superior de la Federación? [724-724]
1: Si
2: No
9: NS

*QUESTION 725 *CODES 825L1
p63) ¿Trabajaba usted en la administración del municipio el año pasado, 2011? [725-725]
1: Si
2: No
9: NS

*QUESTION 726 *NUMBER 826L3 *MIN 0 *MAX 100
Es comun que los gobiernos municipales están inciertos acerca de la probabilidad de una futura auditoria por parte de la Auditoria Superior de la Federación. Las siguientes preguntas se refieren a esas probabilidades para este y los siguientes años:
En una escala de 0 a 100 donde 0 significa que no hay ninguna probabilidad y 100

significa completamente probable

p64) cual cree Usted que es la probabilidad de que su municipio sea auditado por la ASF este año? [726-728]

*QUESTION 729 *NUMBER 829L3 *MIN 0 *MAX 100
p65) cual cree Usted que es la probabilidad de que su municipio sea auditado por la ASF el siguiente año? [729-731]

*QUESTION 732 *NUMBER 832L3 *MIN 0 *MAX 100
Pensando en los próximos tres años, es decir en el 2013, 14, y 15:
En una escala de 0 a 100 donde 0 significa que no hay ninguna probabilidad y 100 significa completamente probable,

p66) ¿Cuál cree usted que es la probabilidad de que su municipio sea auditado por lo menos una vez durante los tres años por la ASF? [732-734]

*QUESTION 735 *NUMBER 835L3 *MIN 0 *MAX 100
p67) ¿Cuál cree usted que es la probabilidad de que su municipio sea auditado dos veces durante los tres años por la ASF? [735-737]

*QUESTION 738 *NUMBER 838L3 *MIN 0 *MAX 100
p68) ¿Cuál cree usted que es la probabilidad de que su municipio sea auditado los tres años por la ASF? [738-740]

*QUESTION 741 *NUMBER 841L3 *MIN 0 *MAX 100
Es común que los gobiernos municipales están inciertos acerca de la probabilidad de una futura auditoria por parte de la Entidad de Fiscalización Estatal. Las siguientes preguntas se refieren a esas probabilidades para este y los siguientes años:
En una escala de 0 a 100 donde 0 significa que no hay ninguna probabilidad y 100 significa completamente probable

p69) ¿Cuál cree Usted que es la probabilidad de que su municipio sea auditado por la EF este año? [741-743]

*QUESTION 744 *NUMBER 844L3 *MIN 0 *MAX 100
p70) ¿Cuál cree Usted que es la probabilidad de que su municipio sea auditado por la EF el siguiente año? [744-746]

*QUESTION 747 *NUMBER 847L3 *MIN 0 *MAX 100
Pensando en los próximos tres años, es decir en el 2013, 14, y 15:
En una escala de 0 a 100 donde 0 significa que no hay ninguna probabilidad y 100 significa completamente probable,

p71) ¿Cuál cree usted que es la probabilidad de que su municipio sea auditado por lo menos una vez durante los tres años por la EF? [747-749]

*QUESTION 750 *NUMBER 850L3 *MIN 0 *MAX 100
p72) ¿Cuál cree usted que es la probabilidad de que su municipio sea auditado dos veces durante los tres años por la EF? [750-752]

*QUESTION 753 *NUMBER 853L3 *MIN 0 *MAX 100
p73) ¿Cuál cree usted que es la probabilidad de que su municipio sea auditado los

tres años por la EF? [753-755]

*QUESTION 756 *CODES 856L1
Modulo: Capacidad del municipio
Pensando en la capacidad del municipio para cumplir con las metas del Fondo para la Infraestructura Social Municipal (FISM), podría por favor manifestar su opinión sobre los siguientes aspectos. Para cada frase dígame si esta totalmente de acuerdo, algo de acuerdo, algo en desacuerdo o totalmente en desacuerdo.

p74) Su ayuntamiento dispone de información actualizada sobre las necesidades en el municipio   [756-756]
1: Totalmente de acuerdo
2: Algo de acuerdo
3: Algo en desacuerdo
4: Totalmente en desacuerdo
9: No sabe/No contesto

*QUESTION 757 *CODES 857L1
p75) Su ayuntamiento dispone de personal especializado para cumplir con los objetivos del Fondo para la Infraestructura Social Municipal (FISM) [757-757]
1: Totalmente de acuerdo
2: Algo de acuerdo
3: Algo en desacuerdo
4: Totalmente en desacuerdo
9: No sabe/No contesto

*QUESTION 758 *CODES 858L1
p76) Su ayuntamiento dispone de suficiente personal para implementar y supervisar las obras con cargo al FISM [758-758]
1: Totalmente de acuerdo
2: Algo de acuerdo
3: Algo en desacuerdo
4: Totalmente en desacuerdo
9: No sabe/No contesto

*QUESTION 759 *CODES 859L1
p77) Su ayuntamiento dispone de mecanismos para evaluar, de forma anual, los resultados las inversiones con cargo al FISM
1: Totalmente de acuerdo
2: Algo de acuerdo
3: Algo en desacuerdo
4: Totalmente en desacuerdo
9: No sabe/No contesto

*QUESTION 760 *CODES 860L1
p78) ¿En el curso de este año, 2012, ha recibido o planea recibir capacitación o asesoría para el desempeño de sus funciones en la administración local? [759-759]
1: Si
2: No
3: Planea recibir
4: No sabe

*QUESTION 761 *CODES 861L1
p79) ¿En el curso de este año, 2012, sabe si el municipio ha impartido, o tiene planeado impartir, cursos de capacitación o asesoría para algunos de sus funcionarios? [760-760]
1: Si
2: No

3: Planea recibir
4: No sabe

*QUESTION 762 *CODES 862L1
p80) ¿En caso de haber recibido, o de planear recibir, cursos de capacitación, de quien espera recibir esa formación? [761-761]
1: Gobierno federal
2: Gobierno estatal
3: Organizaciones no gubernamentales
4: Auditoria Superior de la Federación
5: Otros
6: No Aplica

*QUESTION 763 *ALPHA 863L50 *IF[Q762,5]
p81) Otros [762-811]
