# Supplementary material for: Do federal and state audits increase compliance with a grant program to improve municipal infrastructure (AUDIT study): study protocol for a randomized controlled trial
Source: BMC Public Health. 2014 Sep 3;14:912. doi: 10.1186/1471-2458-14-912 (PMC4175219; doi:10.1186/1471-2458-14-912)
Supplement: Supplementary file 3 — Additional file 3: Survey instrument. (PDF 92 KB) [file 12889_2013_7060_MOESM3_ESM.pdf]

Estimado NOMBRE DEL ENCUESTADO:

Mi nombre es NOMBRE DEL ENCUESTADOR, trabajo para la empresa Data OPM y estamos realizando un proyecto de investigación para la Universidad de Yale. El proyecto esta a cargo de la Dra. Ana De La O y tiene como objetivo entender como los profesionistas que trabajan en el gobierno municipal toman decisiones y perciben a su municipio.

Su experiencia como parte del gobierno municipal es invaluable para el proyecto. ¿Estaría usted dispuesto a completar una encuesta telefónica para este proyecto?

La encuesta toma menos de 20 minutos. Todas sus respuestas (así como su decisión de participar en la encuesta) son confidenciales. Solo los investigadores involucrados en este estudio tendrán acceso a la información que usted provea.

Su participación en este estudio es voluntaria. Usted puede dar por terminada la encuesta en cualquier momento o dejar de contestar algunas preguntas.

Para agradecer su valiosa participación en nuestro estudio, el equipo de investigación le regalara una suscripción de cuatro números a la revista Este País.

Si tiene preguntas acerca de esta encuesta, Usted puede contactar directamente a la investigadora encargada del proyecto al (52 55) 53-51-26-92 o enviar un correo electrónico a la siguiente dirección: ana.delao@yale.edu.

Si desea hablar con alguien ajeno al proyecto para compartir sus preguntas, o para discutir sus derechos como participante de esta encuesta, Usted puede ponerse al contacto con el Comité responsable de cudiar los derechos de entrevistados llamado *Human Subjects Committee* de la universidad de Yale, Box 208010, New Haven, CT 06520-8010, 203-785-4688, human.subjects@yale.edu. Información adicional esta disponible en el siguiente link: http://www.yale.edu/hrpp/participants/index.html

*QUESTION 1 *CODES 101L1
X1) Genero [1-1]
1: Masculino
2: Femenino

*QUESTION 2 *NUMBER 102L2
X2) Edad [2-3]

*QUESTION 4 *ALPHA 104L50
X3) Lugar de nacimiento   [4-53]

*QUESTION 54 *ALPHA 154L50
X4) Grado de estudios   [54-103]

*QUESTION 104 *ALPHA 204L50
X5) Empleo actual   [104-153]

*QUESTION 154 *ALPHA 254L50
X6) Area [154-203]

*QUESTION 204 *ALPHA 304L50
X7) Empleo anterior   [204-253]

*QUESTION 254 *ALPHA 354L50
X8) Correo electrónico [254-303]

*QUESTION 304 *NUMBER 404L20
X9) Teléfonos [304-323]

*QUESTION 324 *CODES 424L1
MODULO: CARRERA PROFESIONAL

P1) ¿Cada gobierno municipal tiene una duración de tres años, en cuantos gobiernos municipales ha trabajado usted a lo largo de su carrera profesional? [324-324]
1: Uno
2: Dos
3: Tres
4: Cuatro
5: Cinco o mas

*QUESTION 325 *CODES 425L1
p2) ¿Alguna vez ha formado usted parte de un gobierno estatal? [325-325]
1: Si
2: No

*QUESTION 326 *CODES 426L1
p3) ¿Alguna vez ha formado usted parte del congreso estatal? [326-326]
1: Si
2: No

*QUESTION 327 *CODES 427L1
p4) ¿Alguna vez ha formado usted parte de un gobierno federal? [327-327]
1: Si
2: No

*QUESTION 328 *CODES 428L1
p5) ¿Alguna vez ha formado usted parte del congreso federal? [328-328]
1: Si, especifique a cual________

2: No

*QUESTION 329 *ALPHA 429L100 *IF[Q328,1]
p5a) especifique a cual? [329-428]

*QUESTION 429 *CODES 529L1
p6) ¿Ocupa usted, o ha ocupado usted, algún cargo en un partido político? [429-429]
1: Si Especifique partido y cargo _________
2: No

*QUESTION 430 *ALPHA 530L100 *IF[Q429,1]
p6a) Especifique partido y cargo _________ [430-529]

*QUESTION 530 *CODES 630L1
p7) ¿Qué actividad profesional espera desempeñar una vez que termine su gestión municipal? LEER OPCIONES [530-530]
1: Trabajar en el próximo gobierno municipal
2: Trabajar en el próximo gobierno estatal
3: Trabajar en el gobierno federal
4: Negocio Propio
5: Otro Especifique____

*QUESTION 531 *ALPHA 631L50 *IF[Q530,5]
p7a) Otro Especifique____ [531-580]

*QUESTION 581 *CODES 681L1
p8a) ¿Qué actividad profesional espera desempeñar una vez que termine su gestión municipal?   Elegir solamente los 3 que más le preocupan de la siguiente lista LEER OPCIONES (MENCION 1) [581-581]
1: Auditoria superior de la federación
2: Empresarios locales
3: Residentes fuera de la cabecera municipal
4: Residentes dentro de la cabecera municipal
5: Presidente Municipal
6: Sindicatos
7: Su partido u organización política
9: No contesto

*QUESTION 582 *CODES 682L1
p8b) ¿A la hora de rendir cuentas sobre su desempeño en el municipio, la opinión de quien le preocupa más? Elegir solamente los 3 que más le preocupan de la siguiente lista LEER OPCIONES (MENCION 2) [582-582]
1: Auditoria superior de la federación
2: Empresarios locales
3: Residentes fuera de la cabecera municipal
4: Residentes dentro de la cabecera municipal
5: Presidente Municipal
6: Sindicatos
7: Su partido u organización política
9: No contesto

*QUESTION 583 *CODES 683L1
p8c) ¿A la hora de rendir cuentas sobre su desempeño en el municipio, la opinión de quien le preocupa más? Elegir solamente los 3 que más le preocupan de la siguiente lista LEER OPCIONES (MENCION 3) [583-583]
1: Auditoria superior de la federación
2: Empresarios locales
3: Residentes fuera de la cabecera municipal

4: Residentes dentro de la cabecera municipal
5: Presidente Municipal
6: Sindicatos
7: Su partido u organización política
9: No contesto

*QUESTION 584 *ALPHA 684L50
p9) De estos 3, ¿Qué opinión es la que más le preocupa? ____ [584-633]

*QUESTION 634 *CODES 734L1
Una vez terminada la actual legislatura municipal, y pensando en sus perspectivas laborales, que tan de acuerdo está con los siguientes: Para cada frase dígame si está totalmente de acuerdo, algo de acuerdo, algo en desacuerdo o totalmente en desacuerdo.
p10) El hecho de haber trabajado en este ayuntamiento mejorará sus perspectivas laborales [634-634]
1: Totalmente de acuerdo
2: Algo de acuerdo
3: Algo en desacuerdo
4: Totalmente en desacuerdo
5: No sabe - No contesto

*QUESTION 635 *CODES 735L1
p11) En su siguiente ocupación, espera recibir mejor salario [635-635]
1: Totalmente de acuerdo
2: Algo de acuerdo
3: Algo en desacuerdo
4: Totalmente en desacuerdo
5: No sabe-No contesto

*QUESTION 636 *CODES 736L1
p12) En su entorno social esta bien considerado el haber sido un servidor público [636-636]
1: Totalmente de acuerdo
2: Algo de acuerdo
3: Algo en desacuerdo
4: Totalmente en desacuerdo
5: No sabe-No contesto

*QUESTION 637 *CODES 737L1
Modulo: Conocimiento del Fondo de Infraestructura Social Municipal

Ahora le voy a preguntar sobre el Fondo de Aportaciones para la Infraestructura Social Municipal (FISM)
De lo que sabe o ha oído ¿Permiten las disposiciones normativas del Fondo de Aportaciones para la Infraestructura Social Municipal (FISM) financiar los siguientes rubros?

p13) Alcantarillado [637-637]
1: Si
2: No
9: NS

*QUESTION 638 *CODES 738L1
p14) Mejoramiento de vivienda [638-638]
1: Si
2: No

9: NS

*QUESTION 639 *CODES 739L1
p15) Caminos rurales [639-639]
1: Si
2: No
9: NS

*QUESTION 640 *CODES 740L1
p16) Kioskos [640-640]
1: Si
2: No
9: NS

*QUESTION 641 *CODES 741L1
p17) Fiestas patronales [641-641]
1: Si
2: No
9: NS

*QUESTION 642 *CODES 742L1
p18) Drenaje y letrinas [642-642]
1: Si
2: No
9: NS

*QUESTION 643 *CODES 743L1
p19) Infraestructura básica de salud [643-643]
1: Si
2: No
9: NS

*QUESTION 644 *CODES 744L1
p20) Remodelación de Iglesias [644-644]
1: Si
2: No
9: NS

*QUESTION 645 *CODES 745L1
p21) Infraestructura básica de educación [645-645]
1: Si
2: No
9: NS

*QUESTION 646 *CODES 746L1
p22) Agua potable [646-646]
1: Si
2: No
9: NS

*QUESTION 647 *CODES 747L1
p23) Programas de Desarrollo Institucional [647-647]
1: Si
2: No
9: NS

*QUESTION 648 *CODES 748L1
p24) ¿Sabe usted si se puede o no gastar los fondos del FISM en obras y acciones sociales que no beneficien directamente a la población en rezago social y pobreza

extrema? [648-648]
1: Si
2: No
9: NS

*QUESTION 649 *CODES 749L1
p25) ¿Tiene su municipio la obligación de publicar, en su órgano local de difusión o página electrónica, informes trimestrales sobre el ejercicio y destino del fondo? [649-649]
1: Si
2: No
9: NS

*QUESTION 651 *CODES 751L1
p27) ¿Está su municipio obligado a informar sobre el ejercicio y destino del FISM a las siguientes instituciones: SEDESOL [651-651]
1: Si
2: No
9: NS

*QUESTION 652 *CODES 752L1
p28) ¿Está su municipio obligado a informar sobre el ejercicio y destino del FISM a las siguientes instituciones: SHCP [652-652]
1: Si
2: No
9: NS

*QUESTION 653 *CODES 753L1
p29) ¿Está su municipio obligado a informar sobre el ejercicio y destino del FISM a las siguientes instituciones: SSP [653-653]
1: Si
2: No
9: NS

*QUESTION 654 *CODES 754L1
p30) ¿Está su municipio obligado a informar sobre el ejercicio y destino del FISM a las siguientes instituciones: SEP [654-654]
1: Si
2: No
9: NS

*QUESTION 655 *CODES 755L1
p31) ¿Está su municipio obligado a informar sobre el ejercicio y destino del FISM a las siguientes instituciones: Gobierno Estatal [655-655]
1: Si
2: No
9: NS

*QUESTION 656 *CODES 756L1
p32) ¿Está su municipio obligado a informar sobre el ejercicio y destino del FISM a las siguientes instituciones: H. Congreso de la Unión [656-656]
1: Si
2: No
9: NS

*QUESTION 657 *CODES 757L1
p33) El Sistema de Formato Único, ¿Es una herramienta de internet creada por el

gobernador estatal para mejorar la gestión del FISM? [657-657]
1: Si
2: No
9: NS

*QUESTION 658 *CODES 758L1
p34) ¿Es necesaria la participación del Comité para la Planeación del Desarrollo Municipal (COPLADEMUN) o, en su defecto, el Consejo de Desarrollo Social Municipal (CDM), para programar las obras e inversiones del fondo en su municipio? [658-658]
1: Si
2: No
9: NS

*QUESTION 659 *CODES 759L1
p35) ¿La participación de los anteriores comités es también necesaria para el seguimiento y evaluación del fondo? [659-659]
1: Si
2: No
9: NS

*QUESTION 660 *CODES 760L1
p36) ¿El gobierno del estado deposita los recursos del FISM en una cuenta bancaria en su municipio de manera anual? [660-660]
1: Si
2: No
9: NS

*QUESTION 662 *CODES 762L1
p37) ¿Tiene el municipio que reportar a la Secretaría de Hacienda y Crédito Público (la SHCP) que áreas del municipio estuvieron encargadas del ejercicio y destino de los recursos del FISM ? [662-662]
1: Si
2: No
9: NS

*QUESTION 663 *NUMBER 763L3 *MIN 0 *MAX 100

Modulo: Prioridades acerca del FISM
Del 100% de los recursos del FISM, ¿que porcentaje considera Usted que se debe gastar en la cabecera municipal,
p39) 1. Cabecera municipal [663-665]

*QUESTION 666 *NUMBER 766L3 *MIN 0 *MAX 100

p40)2. y que porcentaje en el resto de su municipio? [666-668]

*QUESTION 669 *NUMBER 769L3 *MIN 0 *MAX 100
Del 100% de los recursos del FISM, ¿que porcentaje considera Usted que se debe gastar en cada uno de los siguientes tipos de servicios públicos en su municipio?

p41) Servicios como salud, clínicas, drenaje, caminos, y puentes [669-671]

*QUESTION 672 *NUMBER 772L3 *MIN 0 *MAX 100
p42) Servicios como parques, dispensarios, plazas, kioscos y centros comunitarios [672-674]

*QUESTION 675 *NUMBER 775L3 *MIN 0 *MAX 100
p43) Servicios como mejoramiento de vivienda, desayunos escolares, y despensas

[675-677]

*QUESTION 678 *NUMBER 778L3 *MIN 0 *MAX 100
Del 100% de los recursos del FISM, ¿que porcentaje considera Usted que le gustaría a la población que se gastase en cada uno de los siguientes tipos de servicios públicos en su municipio?
p44) Servicios como salud, clínicas, drenaje, caminos, y puentes [678-680]

*QUESTION 681 *NUMBER 781L3 *MIN 0 *MAX 100
p45) Servicios como parques, dispensarios, plazas, kioscos y centros comunitarios [681-683]

*QUESTION 684 *NUMBER 784L3 *MIN 0 *MAX 100
p46) Servicios como mejoramiento de vivienda, desayunos escolares, y despensas [684-686]

*QUESTION 687 *NUMBER 787L3 *MIN 0 *MAX 100
Si el ayuntamiento de un municipio vecino al suyo quisiera ganar las elecciones locales, del 100% de los recursos del FISM, ¿que porcentaje considera Usted que se debería gastar en cada uno de los siguientes tipos servicios públicos en su municipio?

p47) Servicios como salud, clínicas, drenaje, caminos, y puentes [687-689]

*QUESTION 690 *NUMBER 790L3 *MIN 0 *MAX 100
p48) Servicios como parques, dispensarios, plazas, kioscos y centros comunitarios [690-692]

*QUESTION 693 *NUMBER 793L3 *MIN 0 *MAX 100
p49) Servicios como mejoramiento de vivienda, desayunos escolares, y despensas [693-695]

*QUESTION 696 *NUMBER 796L3 *MIN 0 *MAX 100
De los siguientes servicios públicos, ¿qué porcentaje de las necesidades cree Usted que se cubren en la Cabecera municipal?

p50) Agua potable [696-698]

*QUESTION 699 *NUMBER 799L3 *MIN 0 *MAX 100
p51) Drenaje y alcantarillado [699-701]

*QUESTION 702 *NUMBER 802L3 *MIN 0 *MAX 100
p52) Alumbrado público [702-704]

*QUESTION 705 *NUMBER 805L3 *MIN 0 *MAX 100
p53) Pavimentación [705-707]

*QUESTION 708 *NUMBER 808L3 *MIN 0 *MAX 100
De los siguientes servicios públicos, ¿qué porcentaje de las necesidades cree Usted que se cubren en el resto de su Municipio?

p54) Agua potable [708-710]

*QUESTION 711 *NUMBER 811L3 *MIN 0 *MAX 100
p55) Drenaje y alcantarillado [711-713]

*QUESTION 714 *NUMBER 814L3 *MIN 0 *MAX 100
p56) Alumbrado público [714-716]

*QUESTION 717 *NUMBER 817L3 *MIN 0 *MAX 100
p57) Pavimentación [717-719]

*QUESTION 720 *CODES 820L1
Modulo: Auditorias

p58) ¿Ha odio usted hablar de la Entidad de Fiscalización Estatal?   [720-720]
1: Si
2: No
9: NS

*QUESTION 721 *CODES 821L1
p59) ¿Ha odio usted hablar de la Auditoria superior de la federación?   [721-721]
1: Si
2: No
9: NS

*QUESTION 722 *CODES 822L1
p60) ¿Sabe usted si la ASF tiene el poder legal para revisar el manejo, por parte de los municipios, de los fondos del Ramo 33? [722-722]
1: Si
2: No
9: NS

*QUESTION 723 *CODES 823L1
p61) ¿Fue su municipio auditado el año pasado (2011) por la Entidad de Fiscalización Estatal? [723-723]
1: Si
2: No
9: NS

*QUESTION 724 *CODES 824L1
p62) ¿Fue su municipio auditado el año pasado (2011) por la Auditoria Superior de la Federación? [724-724]
1: Si
2: No
9: NS

*QUESTION 725 *CODES 825L1
p63) ¿Trabajaba usted en la administración del municipio el año pasado, 2011? [725-725]
1: Si
2: No
9: NS

*QUESTION 726 *NUMBER 826L3 *MIN 0 *MAX 100
Es comun que los gobiernos municipales están inciertos acerca de la probabilidad de una futura auditoria por parte de la Auditoria Superior de la Federación. Las siguientes preguntas se refieren a esas probabilidades para este y los siguientes años:
En una escala de 0 a 100 donde 0 significa que no hay ninguna probabilidad y 100

significa completamente probable

p64) cual cree Usted que es la probabilidad de que su municipio sea auditado por la ASF este año? [726-728]

*QUESTION 729 *NUMBER 829L3 *MIN 0 *MAX 100
p65) cual cree Usted que es la probabilidad de que su municipio sea auditado por la ASF el siguiente año? [729-731]

*QUESTION 732 *NUMBER 832L3 *MIN 0 *MAX 100
Pensando en los próximos tres años, es decir en el 2013, 14, y 15:
En una escala de 0 a 100 donde 0 significa que no hay ninguna probabilidad y 100 significa completamente probable,

p66) ¿Cuál cree usted que es la probabilidad de que su municipio sea auditado por lo menos una vez durante los tres años por la ASF? [732-734]

*QUESTION 735 *NUMBER 835L3 *MIN 0 *MAX 100
p67) ¿Cuál cree usted que es la probabilidad de que su municipio sea auditado dos veces durante los tres años por la ASF? [735-737]

*QUESTION 738 *NUMBER 838L3 *MIN 0 *MAX 100
p68) ¿Cuál cree usted que es la probabilidad de que su municipio sea auditado los tres años por la ASF? [738-740]

*QUESTION 741 *NUMBER 841L3 *MIN 0 *MAX 100
Es común que los gobiernos municipales están inciertos acerca de la probabilidad de una futura auditoria por parte de la Entidad de Fiscalización Estatal. Las siguientes preguntas se refieren a esas probabilidades para este y los siguientes años:
En una escala de 0 a 100 donde 0 significa que no hay ninguna probabilidad y 100 significa completamente probable

p69) ¿Cuál cree Usted que es la probabilidad de que su municipio sea auditado por la EF este año? [741-743]

*QUESTION 744 *NUMBER 844L3 *MIN 0 *MAX 100
p70) ¿Cuál cree Usted que es la probabilidad de que su municipio sea auditado por la EF el siguiente año? [744-746]

*QUESTION 747 *NUMBER 847L3 *MIN 0 *MAX 100
Pensando en los próximos tres años, es decir en el 2013, 14, y 15:
En una escala de 0 a 100 donde 0 significa que no hay ninguna probabilidad y 100 significa completamente probable,

p71) ¿Cuál cree usted que es la probabilidad de que su municipio sea auditado por lo menos una vez durante los tres años por la EF? [747-749]

*QUESTION 750 *NUMBER 850L3 *MIN 0 *MAX 100
p72) ¿Cuál cree usted que es la probabilidad de que su municipio sea auditado dos veces durante los tres años por la EF? [750-752]

*QUESTION 753 *NUMBER 853L3 *MIN 0 *MAX 100
p73) ¿Cuál cree usted que es la probabilidad de que su municipio sea auditado los

tres años por la EF? [753-755]

*QUESTION 756 *CODES 856L1
Modulo: Capacidad del municipio
Pensando en la capacidad del municipio para cumplir con las metas del Fondo para la Infraestructura Social Municipal (FISM), podría por favor manifestar su opinión sobre los siguientes aspectos. Para cada frase dígame si esta totalmente de acuerdo, algo de acuerdo, algo en desacuerdo o totalmente en desacuerdo.

p74) Su ayuntamiento dispone de información actualizada sobre las necesidades en el municipio [756-756]
1: Totalmente de acuerdo
2: Algo de acuerdo
3: Algo en desacuerdo
4: Totalmente en desacuerdo
9: No sabe/No contesto

*QUESTION 757 *CODES 857L1
p75) Su ayuntamiento dispone de personal especializado para cumplir con los objetivos del Fondo para la Infraestructura Social Municipal (FISM) [757-757]
1: Totalmente de acuerdo
2: Algo de acuerdo
3: Algo en desacuerdo
4: Totalmente en desacuerdo
9: No sabe/No contesto

*QUESTION 758 *CODES 858L1
p76) Su ayuntamiento dispone de suficiente personal para implementar y supervisar las obras con cargo al FISM [758-758]
1: Totalmente de acuerdo
2: Algo de acuerdo
3: Algo en desacuerdo
4: Totalmente en desacuerdo
9: No sabe/No contesto

*QUESTION 759 *CODES 859L1
p77) Su ayuntamiento dispone de mecanismos para evaluar, de forma anual, los resultados las inversiones con cargo al FISM
1: Totalmente de acuerdo
2: Algo de acuerdo
3: Algo en desacuerdo
4: Totalmente en desacuerdo
9: No sabe/No contesto

*QUESTION 760 *CODES 860L1
p78) ¿En el curso de este año, 2012, ha recibido o planea recibir capacitación o asesoría para el desempeño de sus funciones en la administración local? [759-759]
1: Si
2: No
3: Planea recibir
4: No sabe

*QUESTION 761 *CODES 861L1
p79) ¿En el curso de este año, 2012, sabe si el municipio ha impartido, o tiene planeado impartir, cursos de capacitación o asesoría para algunos de sus funcionarios? [760-760]
1: Si
2: No

3: Planea recibir
4: No sabe

*QUESTION 762 *CODES 862L1
p80) ¿En caso de haber recibido, o de planear recibir, cursos de capacitación, de quien espera recibir esa formación? [761-761]
1: Gobierno federal
2: Gobierno estatal
3: Organizaciones no gubernamentales
4: Auditoria Superior de la Federación
5: Otros
6: No Aplica

*QUESTION 763 *ALPHA 863L50 *IF[Q762,5]
p81) Otros [762-811]
